# Lamellipodin-RICTOR Signaling Mediates Glioblastoma Cell Invasion and Radiosensitivity Downstream of EGFR

**DOI:** 10.3390/cancers13215337

**Published:** 2021-10-24

**Authors:** Stefanie Moritz, Matthias Krause, Jessica Schlatter, Nils Cordes, Anne Vehlow

**Affiliations:** 1 OncoRay-National Center for Radiation Research in Oncology, Faculty of Medicine, Technische Universität Dresden, Fetscherstraße 74, PF 41, 01307 Dresden, Germany; Stefanie.Moritz@uniklinikum-dresden.de (S.M.); Nils.Cordes@OncoRay.de (N.C.); 2 Randall Centre of Cell and Molecular Biophysics, New Hunt’s House, Guy’s Campus, King’s College London, London SE1 1UL, UK; matthias.krause@kcl.ac.uk (M.K.); jessica.schlatter@kcl.ac.uk (J.S.); 3 Institute of Radiooncology-OncoRay, Helmholtz-Zentrum Dresden-Rossendorf, Bautzner Landstraße 400, 01328 Dresden, Germany; 4 Department of Radiotherapy and Radiation Oncology, University Hospital Carl Gustav Carus, Technische Universität Dresden, Fetscherstraße 74, PF 50, 01307 Dresden, Germany; 5 German Cancer Consortium (DKTK), Partner Site Dresden, German Cancer Research Center (DKFZ), Im Neuenheimer Feld 280, 69192 Heidelberg, Germany; 6 National Center for Tumor Diseases (NCT), Partner Site Dresden, German Cancer Research Center (DKFZ), Im Neuenheimer Feld 280, 69192 Heidelberg, Germany

**Keywords:** Lamellipodin, glioblastoma, invasion, radiosensitivity, RICTOR, EGFR

## Abstract

**Simple Summary:**

Glioblastoma patients suffer from a poor prognosis with a limited survival of just a few months. Incurability of this tumor mainly results from glioblastoma cell invasiveness as well as therapy resistance. A better understanding of the molecular processes driving aggressive infiltration and resistance to therapeutic intervention would enable the development of new therapeutic approaches. In this study, we identify a so-far-undescribed role of the cytoskeleton protein Lamellipodin in glioblastoma cells. We determined that Lamellipodin essentially mediates glioblastoma invasion, proliferation and radiosensitivity. Our results further identify a new Lamellipodin-RICTOR-EGFR signaling axis enabling glioblastoma radiation survival.

**Abstract:**

Glioblastoma is a tumor type of unmet need despite the development of multimodal treatment strategies. The main factors contributing to the poor prognosis of glioblastoma patients are diverse genetic and epigenetic changes driving glioblastoma persistence and recurrence. Complemented are these factors by extracellular cues mediated through cell surface receptors, which further aid in fostering pro-invasion and pro-survival signaling contributing to glioblastoma therapy resistance. The underlying mechanisms conferring this therapy resistance are poorly understood. Here, we show that the cytoskeleton regulator Lamellipodin (Lpd) mediates invasiveness, proliferation and radiosensitivity of glioblastoma cells. Phosphoproteome analysis identified the epidermal growth factor receptor (EGFR) signaling axis commonly hyperactive in glioblastoma to depend on Lpd. Mechanistically, EGFR signaling together with an interaction between Lpd and the Rapamycin-insensitive companion of mammalian target of rapamycin (RICTOR) jointly regulate glioblastoma radiosensitivity. Collectively, our findings demonstrate an essential function of Lpd in the radiation response and invasiveness of glioblastoma cells. Thus, we uncover a novel Lpd-driven resistance mechanism, which adds an additional critical facet to the complex glioblastoma resistance network.

## 1. Introduction

Glioblastomas are the most devastating type of primary brain tumors in adults and are associated with a dismal prognosis of 12 to 14 months survival [1,2]. Although treatment of these tumors combines maximal surgical resection and fractionated radiochemotherapy, glioblastomas are associated with high mortality [1,3]. Tumor formation, progression and resistance arise from a combination of genetic and epigenetic alterations [1,2,4,5,6] as well as survival advantages initiated by cell adhesion molecules and receptor tyrosine kinases [7,8,9,10]. Gene amplification and mutation of the oncogene epidermal growth factor receptor (EGFR) occur in ~60% of glioblastoma patients [11] and is an indicator for poor prognosis in glioblastoma patients [12,13]. Moreover, the EGFR downstream pathways phosphatidylinositol-4,5-bisphosphate 3-kinase (PI3K)-AKT-mechanistic target of rapamycin kinase (mTOR) signaling and mitogen-activated protein kinase (MAPK) signaling are prominently deregulated and favor therapy resistance in glioblastoma [14,15].

A large network of cytoplasmic molecules transduces intracellular signals and enhances proliferative and migratory properties of glioblastoma cells in response to extracellular matrix-based stimuli. Several studies indicate that the cytoskeleton-associated protein Lamellipodin (Lpd) coordinates actin assembly by the integration of signals from integrin cell adhesion and growth factor receptors [16,17,18,19]. Lpd directly binds to Rac GTPases downstream of growth factor receptors and recruits enabled (Ena)/vasodilator-stimulated phosphoprotein (VASP) and Wiskott–Aldrich syndrome protein (Scar)/WASP-family verprolin-homologous protein (WAVE) protein complexes to the cell membrane [20,21]. Thereby, Lpd modulates the ratio between monomeric and filamentous actin [20,22] and regulates proliferation processes [20,21,23], endocytosis [19,24], cell migration [21,25] and neuronal morphogenesis [16,26,27]. In line with an alteration of these processes in cancer cells, Lpd expression differs in cancer compared to normal tissue. The deletion of the chromosomal region 2q containing the Lpd gene (*RAPH1*—Ras-associated and pleckstrin homology domain-containing protein 1) is commonly observed in head and neck squamous cell cancer, neuroblastoma and lung carcinoma [28,29,30]. While a reduced Lpd expression suggests a tumor suppressor function in osteosarcoma [31], overexpressed Lpd rather points at a function as a predictor for invasiveness and poor prognosis in breast cancer patients; a finding corroborated in preclinical in vitro and in vivo experiments [32,33]. Mechanistically, Lpd drives 3D breast cancer migration via Ena/VASP and Scar/WAVE proteins [33]. In a *Drosophila* glial tumor model, the overexpression of Lpd was capable to promote transformation, hyperproliferation and cell invasion of RAS^v12^-induced glial tumor cells. In this model, Lpd cooperated with Profilin and serum response factor (SRF) signaling rather than with Ena/VASP and Scar/WAVE proteins to promote oncogenic migration [34].

As these observations imply a pathological significance of Lpd in cancer, we investigated the so-far-uncharacterized function of Lpd in highly invasive, proliferative and therapy-resistant glioblastoma. We found that Lpd and EGFR mutually co-operate and Lpd interacts with the rapamycin-insensitive companion of mTOR (RICTOR) consequently critically co-regulating glioblastoma invasion and radiation sensitivity.

## 2. Results

### 2.1. Lamellipodin Promotes Glioblastoma Cell Invasiveness

**Figure 1:**
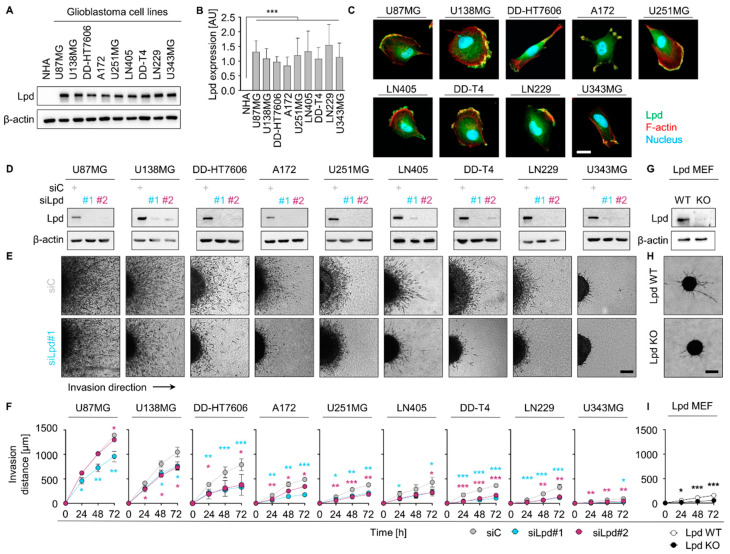
Lamellipodin promotes glioblastoma cell invasion. (**A**) Western blot analysis of Lpd expression in whole-cell lysates of normal human astrocytes (NHA) and indicated human glioblastoma cell cultures. β-actin served as loading control. (**B**) Densitometry analysis of Lpd expression from Western blots in (**A**). Lpd intensity was measured using ImageJ and normalized to β-actin intensity. Results are represented as mean ± SD (*n* = 3, two-sided Student’s *t*-test, *** *p* < 0.001). (**C**) Representative confocal microscopy images of Lpd (green), F-actin (red) and nucleus (blue) in indicated 2D glioblastoma cells cultures plated on type I collagen. Scale bar, 20 µm. (**D**) Western Blot analysis of knockdown efficiency 72 h after transfection of Lpd-specific and control siRNA in indicated glioblastoma cell cultures. β-actin served as loading control. (**E**) Representative phase-contrast images (*t* = 72 h) and (**F**) invasion capacity of control and Lpd-depleted glioblastoma spheroids recorded 24 h, 48 h and 72 h after embedment in 3D type I collagen. Scale bar, 200 µm. Cell lines are ordered by decreasing basal invasive capacity. Results are mean ± SD (*n* = 3, two-sided Student’s *t*-test, * *p* < 0.05, ** *p* < 0.01, *** *p* < 0.001). (**G**) Western blot analysis of Lpd expression in whole-cell lysates from Lpd WT MEF and Lpd KO MEF. β-actin served as loading control. (**H**) Representative phase-contrast images (*t* = 72 h) and (**I**) invasion distance of Lpd WT MEF and Lpd KO MEF spheroids measured 24 h, 48 h and 72 h after embedment in 3D type I collagen. Scale bar: 200 µm. Results are presented as mean ± SD (*n* = 3, two-sided Student’s *t*-test, * *p* < 0.05, ** *p* < 0.01, *** *p* < 0.001). Lpd, Lamellipodin; AU, arbitrary units; si, small interfering RNA; C, control; WT, wild type; KO, knockout; MEF, mouse embryonic fibroblasts.

Extracellular matrix-driven infiltration of tumor cells within the brain parenchyma is a major hallmark of glioblastoma and associated with therapy resistance [35]. Lpd expression was comparatively evaluated in a panel of glioblastoma cell lines against normal human astrocytes (NHA). Surprisingly, we observed a lack of Lpd expression in NHA and cell-line-dependent Lpd protein levels in all tested glioblastoma models (Figure 1A,B). As expected [20], Lpd localized to lamellipodia, which are enriched in F-actin (Figure 1C), suggesting a functional link to glioblastoma invasion.

Next, we evaluated the function of Lpd for glioblastoma invasiveness using a 3D spheroid invasion assay. While the tested glioblastoma cell lines demonstrated varying invasion capacities into three-dimensional collagen type I matrix, Lpd knockdown generally reduced invasion in comparison to controls (Figure 1D,E and Figure S1A). Reduction of invasion distance ranged from 6% in U87MG to 75% in U343MG (Figure 1F and Figure S1B). As a proof of principle of our experimental setup, we also tested invasiveness of Lamellipodin wildtype (Lpd WT) relative to Lamellipodin knockout (Lpd KO) mouse embryonic fibroblasts (MEF) which displayed comparable reduction in invasiveness (Figure 1G–I). Altogether, our data suggest that Lpd significantly contributes to glioblastoma cell invasiveness.

### 2.2. Lamellipodin Facilitates Radiation Survival and Promotes Proliferation of Glioblastoma Cells

**Figure 2:**
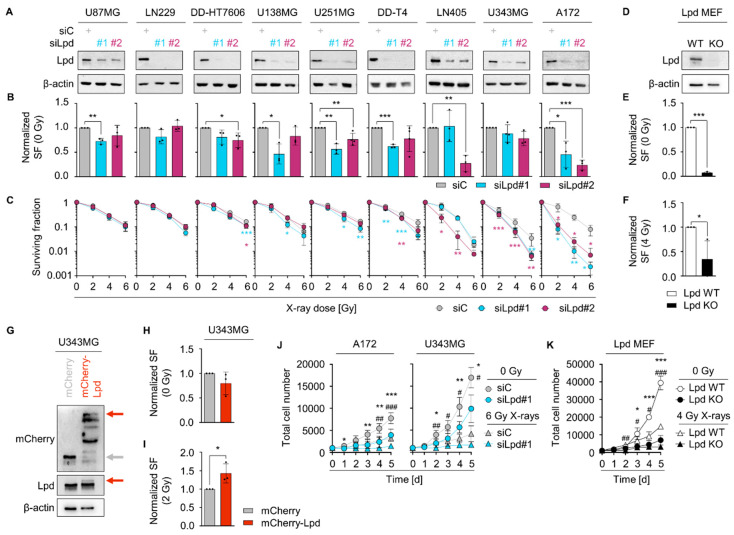
Lamellipodin regulates radiosensitivity and proliferation of glioblastoma cells. (**A**) Western Blot analysis of knockdown efficiency after transfection of Lpd-specific and control siRNA in indicated glioblastoma cell cultures. β-actin served as loading control. (**B**) Surviving fraction (SF) at 0 Gy relative to control knockdown and (**C**) clonogenic radiation survival upon 2, 4 and 6 Gy irradiation of Lamellipodin-depleted glioblastoma cell cultures after cell-line-dependent incubation times (7–15 days). (**D**) Western blot analysis of Lpd and β-actin expression in whole-cell lysates from Lpd WT MEF and Lpd KO MEF showing knockout efficiency. (**E**) Normalized surviving fraction at 0 Gy and (**F**) 4 Gy of Lpd KO MEF compared to Lpd WT MEF. (**G**) Representative Western blot analysis of transient mCherry-empty and mCherry-Lpd vector expression in U343MG. (**H**) Normalized surviving fraction at 0 Gy and (**I**) 2 Gy of mCherry-Lpd-expressing U343MG cells relative to mCherry-empty controls after 10 days. (**J**) Total cell number indicating proliferation of non-irradiated (circle) or 6 Gy-irradiated (triangle) A172 and U343MG cells after siRNA-mediated knockdown of Lamellipodin or control knockdown recorded at days 0 to 5. (**K**) Proliferation (total cell number) of non-irradiated (circle) and 4 Gy irradiated (triangle) Lpd WT MEF and Lpd KO MEF. All results show mean ± SD (*n* = 3–4, two-sided Student’s *t*-test, */# *p* < 0.05, **/## *p* < 0.01, ***/### *p* < 0.001). * indicates *p*-values of non-irradiated and # indicates *p*-values of irradiated conditions in (**J**,**K**). si, small interfering RNA; C, control; Lpd, Lamellipodin; SF, surviving fraction; WT, wild type; KO, knockout; MEF, mouse embryonic fibroblasts.

As Lpd is implicated in cell proliferation control [23], we explored the role of Lpd in glioblastoma cell radiosensitivity (Figure 2A–C). Lpd knockdown decreased the colony-forming capacity of unirradiated cells (basal survival) in more than half of the tested cell lines in comparison to controls (Figure 2B and Figure S2A). Upon exposure to X-rays and Lpd silencing, we detected an additive radiosensitization in seven out of nine Lpd-depleted glioblastoma cell lines (Figure 2C and Table S1), a finding corroborated in MEF (Figure 2D–F and Figure S2B). In line with the previous results, overexpression of mCherry-tagged Lamellipodin (mCherry-Lpd) improved radiation survival of U343MG cells relative to mCherry controls (mCherry) (Figure 2G–I). In addition to clonogenicity, we analyzed the proliferative capacity of U343MG and A172 glioblastoma cells and found a delayed proliferation as well as increased doubling times upon Lpd depletion relative to controls (Figure 2J and Figure S2C). X-ray irradiation enhanced this effect emphasizing a specific function of Lpd in the glioblastoma radioresponse (Figure 2J and Figure S2D). In agreement, Lpd KO MEFs compared to Lpd WT MEFs also showed a diminished proliferation and increased doubling time (Figure 2K and Figure S2D). We further tested whether the decreases in clonogenic survival and proliferation are connected to apoptotic cell death. However, no induction of apoptosis was observed in glioblastoma cells (Figure S2E). Taken together, our findings suggest a critical role of Lpd in glioblastoma cell radioresistance and proliferation.

### 2.3. Lamellipodin Knockdown Enhances Glioblastoma Radiosensitivity via the EGFR-MAPK Signaling Axis

**Figure 3:**
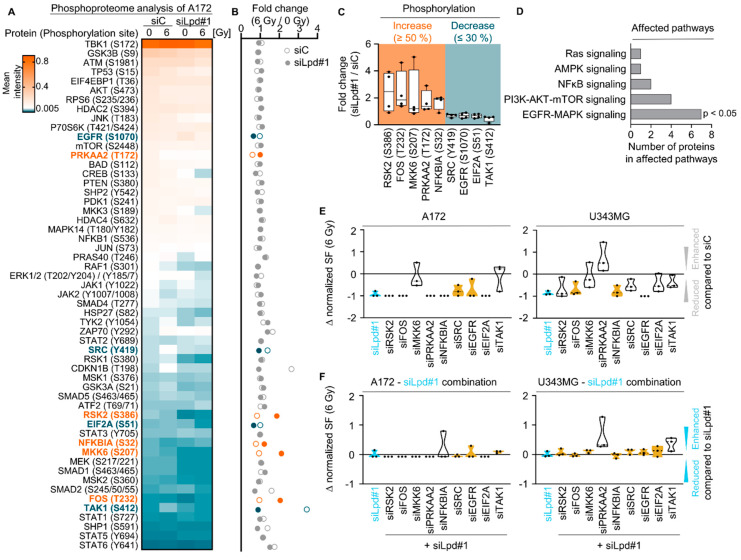
Lamellipodin promotes glioblastoma radiosensitivity via the EGFR-MAPK signaling axis. (**A**) Phosphoproteome analysis showing mean intensity of 55 indicated phosphorylation sites of Lpd or control depleted A172 cell cultures 1 h after 0 Gy or 6 Gy plotted as heat map (*n* = 4). Corresponding data are provided in Table S2. (**B**) Fold change in phosphorylation calculated from mean intensity from (**A**) normalized to 0 Gy. Alterations in phosphorylation (fold change) upon Lpd knockdown and 6 Gy compared to control knockdown are categorized as follows: increase ≥ 50% in orange, decrease ≤ 30% in blue. Corresponding data are provided in Table S3. Data are presented as mean (*n* = 4). (**C**) Identified proteins showing at least 50% increase or 30% decrease in phosphorylation site changes upon Lpd knockdown and 6 Gy X-ray in comparison to controls from (**B**). (**D**) Affected pathways with altered protein phosphorylation sites after Lpd relative to controls from (**C**). Enrichment of proteins in signaling pathways is indicated with *p* < 0.05 (Fischer’s exact test). (**E**) Normalized surviving fraction at 6 Gy upon knockdown of the indicated proteins subtracted by control knockdown (∆ value) showing differential effects on the surviving fraction of A172 and U343MG cells. Data were normalized to the surviving fraction at 6 Gy of the control knockdown. (**F**) Differences in radiosensitizing effects shown by Δ values of normalized surviving fraction 6 Gy upon knockdown of the indicated proteins in combination with Lpd knockdown subtracted by Lpd knockdown of A172 and U343MG cells. ∆ value defines altered survival compared to survival of siC (**E**) or siLpd#1 (**F**) and 6 Gy treated cells: <0 reduced survival, 0 unchanged survival, >0 increased survival. Yellow color display equal survival at 6 Gy comparable to Lamellipodin knockdown, whereas white color marks a diverging survival ∆ value. Data are represented as mean ± SD (*n* = 3, two-sided Student’s *t*-test, *n*.s., not significant, * *p* < 0.05, ** *p* < 0.01, *** *p* < 0.001). si, small interfering RNA; C, control; Lpd, Lamellipodin; SF, surviving fraction.

To get insights into the mechanism, we characterized the signal transduction pathways altered by Lpd silencing during the radioresponse of glioblastoma cells. To do this, we performed a phosphoproteome analysis of 55 phosphorylation sites involved in common signaling pathways (e.g., EGFR, AKT serine/threonine kinase (AKT), MAPK and others) in unirradiated and irradiated Lpd-depleted A172 cells (Tables S2 and S3), which revealed the highest radiosensitization upon Lpd knockdown (Figure 2C). Figure 3A shows a heat map analysis revealing the changes in protein phosphorylation across the different treatment conditions (Figure 3A). A further comparison of radiation-induced phosphorylation site changes upon Lpd versus control knockdown revealed nine affected phosphorylation sites (Figure 3B,C). Deregulated proteins mainly mapped to key molecular mechanisms in glioblastoma such as Ras, PI3K-AKT-mTOR and EGFR-MAPK signaling (Figure 3D). Intriguingly, modifications in proteins of the EGFR-MAPK signaling axis, e.g., Fos proto-oncogene, AP-1 transcription factor subunit (FOS), EGFR, eukaryotic translation initiation factor 2 subunit alpha (EIF2A), MKK6, ribosomal protein S6 kinase A3 (RSK2), SRC proto-oncogene, non-receptor tyrosine kinase (SRC) and mitogen-activated protein kinase kinase kinase 7 (TAK1), were significantly overrepresented in Lpd depleted cells (Figure 3D).

To discover the functional impact of these proteins for the radiosensitizing effects exhibited upon Lpd knockdown, we conducted a knockdown screen with these hits and evaluated basal and clonogenic radiation survival of A172 and U343MG cells. In both glioblastoma cell lines, depletion of FOS, NFKB inhibitor alpha (NFKBIA) and EGFR resulted in similar basal clonogenic and clonogenic radiation survival compared to Lpd depletion (Figure 3E and Figure S3A–D). In addition, equal degrees of cytotoxicity and radiosensitization were reached for single Lpd knockdown compared to combined knockdown of Lpd with either RSK2, FOS, MKK6, NFKBIA, SRC, EGFR or EIF2A (Figure 3F and Figure S4A–D). Taken together, our results suggest that Lpd mediates the glioblastoma radioresponse via the EGFR-MAPK-pathway.

**Figure 4:**
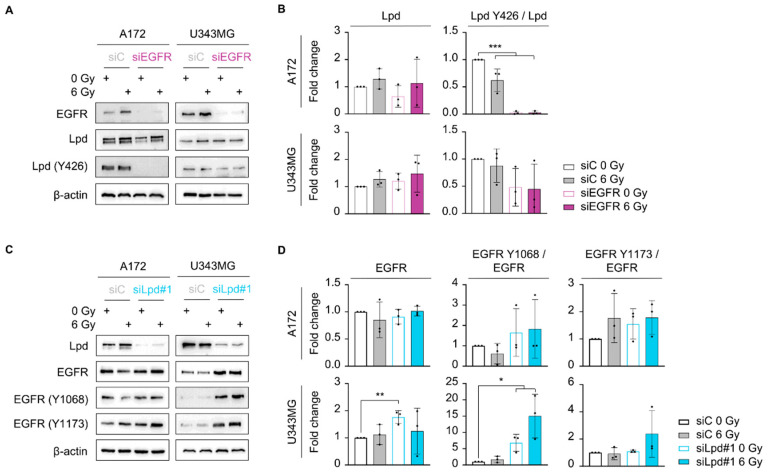
The EGFR-Lpd interplay is cell-line-dependent. (**A**) Representative immunoblot of EGFR, Lpd, phosphorylated Lpd (Y426) and β-actin in A172 and U343MG upon EGFR knockdown and 1 h after irradiation. (**B**) Densitometric analyses showing changes in Lpd expression and phosphorylation from (**A**). Fold changes of Lpd levels are calculated by normalization to β-actin. Fold changes of phosphorylated Lpd Y426 are determined by normalization to total Lpd expression. All protein levels were related to the corresponding β-actin. Fold changes are relative to control-treated and unirradiated samples. (**C**) Representative Western blot from Lpd-silenced A172 and U343MG showing total and phosphorylated EGFR 1 h post-X-ray irradiation. β-actin served as loading control. Representative Western blots are shown. (**D**) Densitometry of EGFR from (**C**). Fold changes of EGFR levels are calculated by normalization to β-actin. Fold changes of phosphorylated EGFR are calculated by normalization to total EGFR expression. All protein levels were related to the corresponding β-actin. Fold changes are relative to control-treated and unirradiated samples. All results are mean ± SD (*n* = 3, two-sided Student’s *t*-test, * *p* < 0.05, ** *p* < 0.01, *** *p* < 0.001). si, small interfering RNA; C, control; Lpd, Lamellipodin.

To clarify the hierarchical functionality of the Lpd-EGFR interplay further, we performed Western blot analysis of EGFR and Lpd expression as well as EGFR Y1068 and Lpd Y426 phosphorylation in the glioblastoma cell line panel (Figure S5). Intriguingly, A172 showed the highest phosphorylation of EGFR and Lpd, suggesting activation of EGFR signaling in these cells. In line with these results, EGFR knockdown reduced Lpd Y426 phosphorylation in both cell lines, with a stronger decrease in phosphorylated Lpd Y426 in A172 cells (Figure 4A,B). Conversely, knockdown of Lpd strongly increased EGFR levels in U343MG and enhanced EGFR phosphorylation at Y1068 and Y1173 in U343MG and A172, albeit to a lower extent (Figure 4C,D). Thus, our data suggest an interdependency of EGFR and Lpd in the glioblastoma radioresponse, whereby EGFR exerts a positive effect on Lpd phosphorylation and Lpd negatively regulates EGFR levels and phosphorylation.

### 2.4. Lamellipodin Forms a Protein Complex with RICTOR

**Figure 5:**
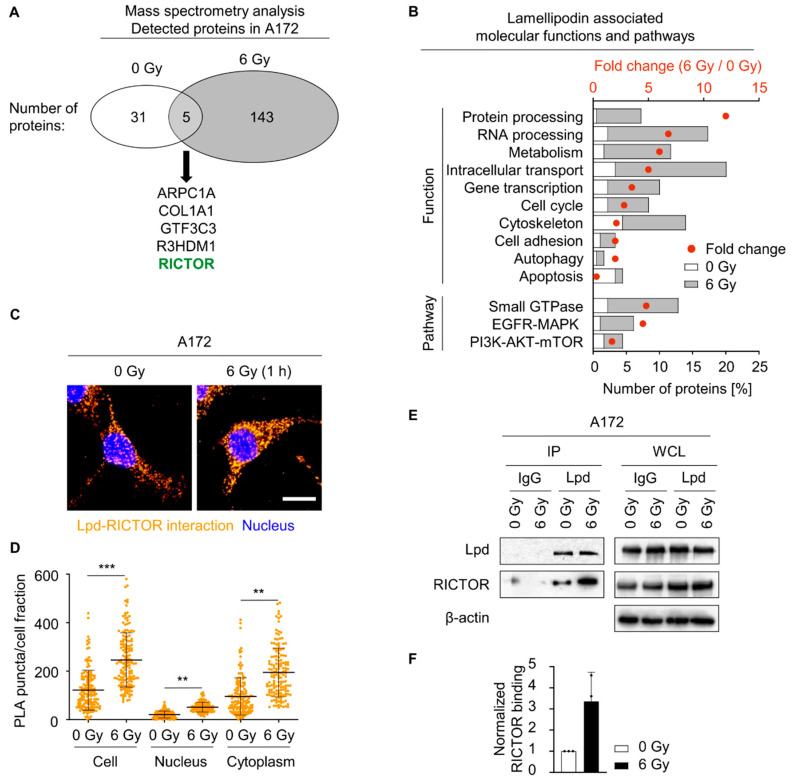
Lamellipodin forms a protein complex with RICTOR. (**A**) Number of detected proteins in unirradiated and 6 Gy-irradiated A172 cells following immunoprecipitation 1 h post-irradiation and mass spectrometry analysis (*n* = 2). All identified proteins are provided in Tables S4 and S5. (**B**) Categorization of Lpd interactome into molecular functions and pathways using Reactome and KEGG pathway classification. Comparative analysis of affected pathways upon 0 Gy and 6 Gy was done by calculation of fold change. (**C**) Proximity ligation assay showing interaction of Lpd and RICTOR (orange) in A172 1 h after 0 Gy or 6 Gy X-rays. Scale bar: 20 µm. (**D**) Quantification of proximity ligation puncta in whole cell, nucleus and cytoplasm from (**C**) using Fiji. Results are presented as mean ± SD (*n* = 3, two-sided Student’s *t*-test, ** *p* < 0.01, *** *p* < 0.001). (**E**) Representative immunoprecipitation of Lpd and non-specific control IgG. Immunoblot showing Lpd and RICTOR in copreciptates and whole-cell lysates from unirradiated and irradiated A172 cells. (**F**) Densitometric analyses presenting relative RICTOR binding from (**E**). Fold changes are calculated by normalization to Lpd and 0 Gy according to representative Western blot. Data are shown as mean ± SD (*n* = 3). Lpd; Lamellipodin; PLA, proximity ligation assay; IP, immunoprecipitation; WCL, whole cell lysate, IgG, immunoglobulin G.

To unravel the so-far unexplored molecular mechanism linking Lpd to glioblastoma cell radiosensitivity, we evaluated the Lpd interactome using mass spectrometry. Mass spectrometry analysis identified 36 Lpd interacting proteins in unirradiated cells (Figure 5A and Table S4) and 148 in irradiated cells (Figure 5A and Table S5). Only 3% of these identified proteins, including Actin-related protein 2/3 complex subunit 1A (ARPC1A), collagen type I alpha 1 chain (COL1A1), general transcription factor IIIC subunit 3 (GTF3C3), R3H domain containing 1 (R3HDM1) and RICTOR, bound Lpd under both unirradiated and irradiated conditions (Figure 5A, Tables S4 and S5). This diversity in interaction partners prompted us to map the Lpd interactome to signaling pathways and associated protein functions. In unirradiated glioblastoma cells, Lpd mainly interacted with proteins involved in intracellular transport and cytoskeleton organization (Figure 5B). Irradiation, however, shifted the spectrum towards interaction partners regulating protein and RNA processing as well as metabolism (Figure 5B). Regarding signal transduction, many of the interacting proteins mapped to frequently deregulated signaling pathways in glioblastoma such as the EGFR-MAPK, PI3K-AKT- mTOR and Small GTPase signaling hubs; a finding in line with our phosphoproteome analysis (Figure 5B).

Among the five overlapping interacting partners was RICTOR, which caught our attention due to its molecular connection to the majority of Lpd associated functions (Figure S6A), EGFR-related signaling, and glioblastoma radioresistance [9,36,37]. To confirm its interaction with Lpd, we performed a proximity ligation assay and immunoprecipitation in unirradiated and irradiated glioblastoma cells and observed a basal Lpd-RICTOR interaction, which increased upon X-ray irradiation in the nucleus of U343MG and A172 and in the cytoplasm of A172 (Figure 5C–F and Figure S6B–E). Taken together, our data suggest a hitherto undescribed Lpd-RICTOR interaction and an involvement of both proteins in glioblastoma cell radiosensitivity.

### 2.5. Lamellipodin and RICTOR Jointly Regulate Glioblastoma Radiosensitivity and Invasion

**Figure 6:**
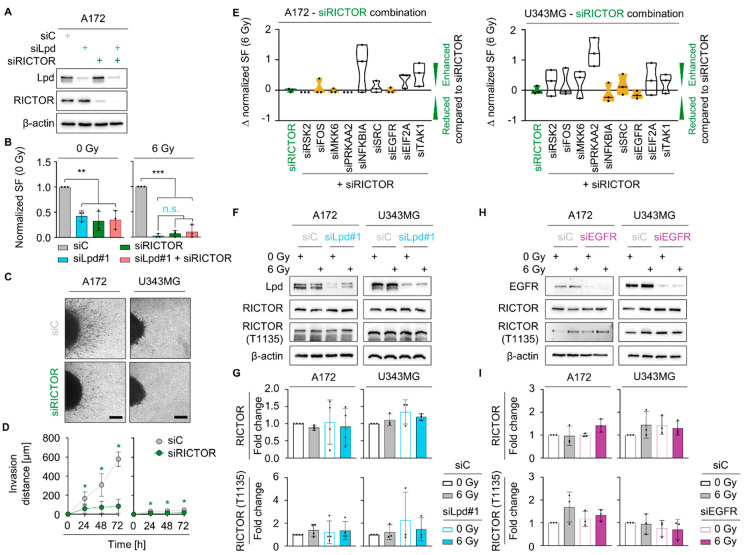
Lamellipodin and RICTOR jointly regulate invasion and radiosensitivity in glioblastoma cells. (**A**) Representative Western blot and (**B**) normalized surviving fraction of non-irradiated and 6 Gy irradiated A172 cells upon Lpd and RICTOR knockdown. (**C**) Representative images of spheroids in 3D collagen I matrix 72 h after seeding. (**D**) Invasion at 24 h, 48 h and 72 h of control and RICTOR siRNA treated A172 and U343MG. (**E**) Differences in radiosensitizing effects shown by Δ values of normalized surviving fraction 6 Gy upon knockdown of the indicated proteins subtracted by RICTOR knockdown in A172 and U343MG cells. ∆ value describes an altered survival relative to the survival of siRICTOR and 6 Gy treated cells: <0 reduced survival, 0 equal survival, >0 increased survival. Yellow color displays similar survival at 6 Gy comparable to RICTOR knockdown, whereas white color mark a differing survival. (**F**) Western blots of Lpd, RICTOR, phospho-RICTOR T1135 and β-actin in Lpd silenced A172 and U343MG cells 1 h after 0 Gy and 6 Gy irradiation. (**G**) Densitometry of RICTOR and phosphorylated RICTOR T1135 from (**F**). Fold changes of RICTOR levels are calculated by normalization to β-actin. Fold changes of phosphorylated RICTOR are determined by normalization to total RICTOR expression. All protein levels are related to the corresponding β-actin. Fold changes are relative to control-treated and unirradiated samples. (**H**) Representative immunoblot showing RICTOR and RICTOR T1135 upon EGFR knockdown and 1 h after irradiation in A172 and U343MG. Actin served as loading control. (**I**) Fold changes of total RICTOR and phosphorylated RICTOR T1335 calculated from (**H**). Fold changes of RICTOR levels are calculated by normalization to β-actin. Fold changes of phosphorylated RICTOR are determined by normalization to total RICTOR expression. All protein levels are related to the corresponding β-actin. Fold changes are relative to control treated and unirradiated samples. All results are presented as mean ± SD (*n* = 3–4, two-sided Student’s *t*-test, *n*.s., not significant, * *p* < 0.05, ** *p* < 0.01, *** *p* < 0.001). si, small interfering RNA; C, control; Lpd, Lamellipodin; SF, surviving fraction.

Since we identified an interaction between Lpd and RICTOR, we questioned whether RICTOR and Lpd share a common signaling axis. Analysis of this functional relevance by clonogenic survival assays revealed a superimposable outcome for basal and radiation survival upon single or combined depletion of Lpd and RICTOR relative to controls (Figure 6A,B and S7A–C). Reduction of RICTOR expression moreover diminished invasion by 77% in A172 and 61% in U343MG, which is comparable to the invasion reduction upon Lpd knockdown alone, suggesting concordant functionality of RICTOR in Lpd-regulated radioresistance and invasion (Figure 6C,D and Figure S7D). Cytoscape analysis predicted RICTOR as part of an interactive network comprising most of the identified proteins associated with Lpd and the EGFR-MAPK cascade (Figure S8A). By silencing RICTOR together with the network components, we detected comparable cytotoxicity and radiosensitization of sole RICTOR knockdown and when combined with EGFR in A172 and U343MG cell lines (Figure 6E and Figure S8B,C). To elucidate the dependency of RICTOR on Lpd and the EGFR, we examined the expression and phosphorylation of RICTOR after Lpd and EGFR knockdown (Figure 6F–I). Intriguingly, RICTOR expression and T1135 phosphorylation associated with activation of its downstream signaling pathway remained unaffected by Lpd and EGFR knockdown.

**Figure 7:**
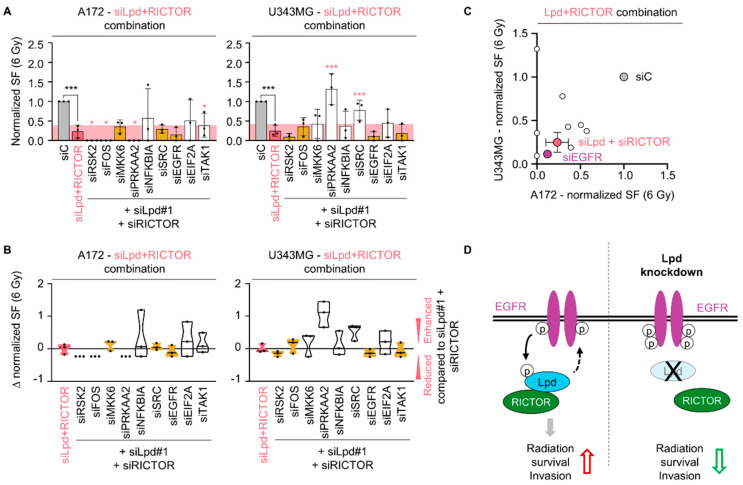
Lamellipodin and RICTOR jointly promote glioblastoma radiation survival via EGFR. (**A**) Normalized clonogenic radiation survival at 6 Gy of A172 and U343MG cells upon siRNA-specific triple knockdown of Lamellipodin, RICTOR and indicated proteins. Yellow columns indicate a surviving fraction comparable to double Lpd and RICTOR knockdown, whereas white columns indicate a differing survival. The pink area represents the normalized surviving fraction upon 6 Gy and Lpd and RICTOR silencing. (**B**) Differences in radiosensitizing effects shown by Δ values of normalized surviving fraction 6 Gy upon knockdown of the indicated proteins subtracted by double Lpd and RICTOR knockdown in A172 and U343MG cells. ∆ value is defined as survival compared to the survival of siLpd and siRICTOR and 6 Gy treated cells: <0 reduced survival, 0 equal survival, >0 increased survival. Yellow color illustrates survival at 6 Gy comparable to Lpd and RICTOR knockdown, whereas white color demonstrates a distinct survival. All results are presented as mean ± SD (*n* = 3, two-sided Student’s *t*-test, siLpd + siRICTOR/siC *** *p* < 0.001, siRNA/siLpd + siRICTOR * *p* < 0.05, ** *p* < 0.01, *** *p* < 0.001). (**C**) Normalized surviving fraction at 6 Gy of A172 and U343MG shown in (**A**). (**D**) Graphical scheme summarizing the interaction of Lamellipodin with RICTOR and the regulation of radiosensitivity and invasion in glioblastoma cells through interplay with the EGFR signaling hub. EGFR exerts a positive effect on Lpd phosphorylation. Conversely, Lpd negatively regulates EGFR levels and phosphorylation. This interplay is cell-line-dependent and may be modulated by basal EGFR activity. Lpd further binds to RICTOR facilitating survival and invasion. Lpd silencing dysregulates EGFR phosphorylation, renders sensitivity to X-rays and reduces invasion. si, small interfering RNA; SF, surviving fraction.

Furthermore, a triple knockdown approach for Lpd, RICTOR and the screen components showed cell-line-dependent effects on basal survival and radiosensitivity (Figure 7A,B and Figure S8D). The combination of Lpd, RICTOR and EGFR induced a similar radiosensitizing effect to that observed for Lpd and RICTOR double knockdown in both cell lines (Figure 7C). Thus, our results suggest that Lpd, RICTOR and EGFR lie within the same signaling axis for the regulation of glioblastoma cell radiosensitivity. Taken together, these data indicate a novel regulatory network between Lpd, RICTOR and EGFR in glioblastoma that jointly determines cell fate upon X-ray irradiation (Figure 7D).

## 3. Discussion

Understanding the fundamental mechanisms driving glioblastoma therapy resistance and invasion may pave the way for more effective therapeutic approaches. Here, we describe that the cytoskeleton regulator Lpd (i) mediates invasion, (ii) is essentially involved in clonogenic radiation survival as well as cell proliferation and (iii) interacts with RICTOR downstream of EGFR to co-control radiosensitivity of glioblastoma cells as shown in Figure 7D.

Lpd is primarily implicated in the regulation of fundamental cellular processes such as cell growth and migration shown for a number of cancer types [16,33,34,38]. Our study expands this knowledge by showing that Lpd regulates invasive movement of glioblastoma cells in 3D collagen type I matrix. While Lpd expression is higher in basal breast cancer cell lines in comparison to luminal breast cancer cell lines and correlates with their invasive capacity and patient outcome [33], we observed that compared to normal human astrocytes, Lpd was highly expressed in all investigated glioblastoma cells independent of their ability to invade 3D collagen I matrix. This diverging Lpd expression pattern in glioblastoma and breast cancer may indicate a tissue-specific function of Lpd. To further expand these findings, an in-depth analysis of Lpd expression in glioblastoma patient samples with regard to its functional and predictive value is warranted.

Owing to our increased understanding that proteins and enzymes serve in different networks in a context-dependent manner, invasion and therapy resistance partly share similar regulatory molecules that may allow glioblastoma cells to evade cell death upon the use of conventional therapies [39]. While the body of literature about proteins connecting invasion and therapy resistance is limited, focal adhesion- and cytoskeleton-associated proteins such as focal adhesion kinase (FAK) and synemin (SYNM) have been described to influence both processes [40,41,42,43]. Intriguingly, our findings add the actin regulator Lpd to the molecular circuitry controlling the cellular response to X-ray irradiation and invasion. Our data show that Lpd impacts invasion and radiation sensitivity to a varying extent in different glioblastoma cell lines reflecting the cellular heterogeneity and molecular complexity of glioblastoma. The plethora of factors creating this discrepancy may cover genetic mutations, epigenetic modifications and diverse escape mechanisms, represented in many studies on characterization of glioblastoma subtypes [5,11,44]. Further in-depth verification in orthotopic mouse models and even clinical samples are warranted to gain functional knowledge of Lpd in glioblastoma.

By means of phosphoproteome arrays, we shed more light on the signaling networks dependent on Lpd in human glioblastoma cells. In addition to deregulated phosphorylation of the well-known glioblastoma driver EGFR [5], we mainly identified deregulated members of the PI3K-AKT-mTOR pathway, MAPK pathway as well as small GTPase signaling upon X-ray irradiation. These results are in line with studies demonstrating that these signaling pathways are interconnected by complex crosstalk mediated by EGFR [45,46] and a physical interaction of Lpd with EGFR [19]. Interestingly, A172 cells showed the highest EGFR and Lpd phosphorylation among the investigated glioblastoma cell lines. In line with this, EGFR knockdown most severely decreased Lpd Y426 phosphorylation in A172 cells, indicating a dependence of both molecules, which might be mediated by SRC, a kinase acting downstream of the EGFR [47,48] driving phosphorylation of Lpd Y426 [16]. In contrast, Lpd phosphorylation in U343MG cells may rely on other unknown signaling mechanisms in addition to the EGFR network. Moreover, U343MG and A172 cells show a higher EGFR phosphorylation upon Lpd knockdown. This may be either linked to a cellular compensation mechanism associated with the adaptor function of Lpd or to Lpd’s described function in EGFR endocytosis, which in turn affects EGFR downstream signaling [16,19]. Furthermore, growth factor receptor recycling is known to control migration and proliferation [49,50] suggesting a deregulated EGFR signaling as a basis for increased radiosensitivity upon Lpd knockdown. Nonetheless, supplementary studies are required to clarify the functional consequences of this observation.

Additional reports describe an interaction of Lpd with the small GTPase and MAPK pathway members Ras and Rac [16,21,51]. Lpd predominantly binds to PI(3,4)P_2_ phospholipids, products of the PI3K pathway, and may compete with AKT, which also binds to PI(3,4)P_2_ [38,52]. It is therefore reasonable that Lpd serves as a mediator between EGFR and other downstream signaling networks. Interestingly, EGFR-MAPK and PI3K-AKT-mTOR signaling is hyperactivated in glioblastoma by EGFR amplification, mutation of the MAPK pathway inhibitor Neurofibromin 1 and loss of the phosphatase and tensin homolog (PTEN) [5,11,53] and is associated with resistance to radiation therapy in glioblastoma [14]. Another important facet in this context is the expression of mutated forms of the EGFR and that molecular-targeted therapies against mutated EGFR failed so far to improve glioblastoma patient overall survival [54,55]. Future studies will discriminate between mechanisms associated with normal and mutated EGFR and the interplay with Lpd.

In addition, our study discovered a novel interaction of Lpd with RICTOR, a regulator of glioblastoma radiosensitivity [9] and chemoresistance in tumors with mutated EGFR by facilitating NFκB signaling [37,56]. Intriguingly, we neither found a dependency of RICTOR expression nor phosphorylation on EGFR and Lpd. However, RICTOR activation is linked to resistance to receptor tyrosine kinase inhibitors [57,58] and RICTOR interacts with EGFR independent of its kinase activity in other tumor entities [59]. Our finding on the Lpd–RICTOR interaction is consistent with several studies demonstrating mTOR independent protein complexes of RICTOR with Cullin-1, integrin-linked kinase (ILK) and protein kinase Cζ (PKCζ) regulating migration, metastasis, survival and protein degradation [60,61,62,63]. In accordance, Lpd and RICTOR knockdown decrease radiosensitivity and invasion to a similar extent to Lpd knockdown alone. In line with these results, previous work shows that RICTOR and Lpd are regulators of the actin cytoskeleton [20,22,64,65]. Lpd recruits Ena/VASP and Scar/WAVE proteins to the leading edge for driving cell migration [20,21,22,33]. RICTOR-mTORC2 affect actin polymerization by PKC- and Rac-GEF-Tiam1-signaling [64,66]. Furthermore, shRNA-mediated RICTOR knockdown increased the WAVE2 complex recruitment to the basal membrane of HL-60 cells, inducing the formation of larger leading edges [67]. Consequently, Lpd and RICTOR may interconnect with the Ena/VASP or Scar/WAVE complex to facilitate invasion in glioblastoma. The differential responses of A172 and U343MG in radiosensitivity after loss of the seven proteins studied in combination with Lpd and RICTOR knockdown additionally suggest that the Lpd-RICTOR-dependent regulation of these signaling pathways is cell-line-dependent. Yet, the commonality of EGFR in radiosensitivity induced by knockdown of Lpd and RICTOR indicates that the Lpd-RICTOR-EGFR signaling pathway is a fundamental mechanism in glioblastoma.

In summary, our study pinpoints the complexity of the cellular response to X-rays by integration of the cytoskeleton-regulating protein Lpd. Mechanistically, we provide information about Lpd interacting with RICTOR as an important determinant downstream of EGFR signaling and thus demonstrate an additional facet of how a cytoskeletal protein takes part in the concerted actions evolving the hallmarks of cancer.

## 4. Materials and Methods

### 4.1. Cell Lines

Clonetics™ normal human astrocytes (NHA) (Lonza, Basel, Switzerland) were cultured in astrocyte basal medium (ABM) 15 mL fetal bovine serum (FBS), 5 mL L-Glutamine, 0.5 mL Gentamicin Sulfate Amphotercin-B (GA-1000), 0.5 mL Ascorbic Acid, 0.5 mL recombinant human epidermal growth factor (rhEGF), 1.25 mL recombinant human Insulin (all from Lonza) at 37 °C with 5% CO_2_. A172, LN229, U87MG, U251MG and U138MG (American Type Culture Collection—ATCC), DD-HT7607 and DD-T4 (kindly provided by A. Temme, Technische Universität Dresden, Dresden, Germany) were cultured in Dulbecco’s Modified Eagle Medium (DMEM, Thermo Fisher Scientific, Waltham, MA, USA), 10% FBS (PAN-Biotech, Aidenbach, Germany) and 1% non-essential amino acids (NEAA, Thermo Fisher Scientific) at 37 °C with 8.5% CO_2_ and humidified atmosphere. U343MG (kindly provided by A. Temme, Technische Universität Dresden, Germany) were grown in Basal Medium Eagle (BME, Thermo Fisher Scientific), supplemented with 10% FBS (PAN-Biotech), 1% NEAA (Thermo Fisher Scientific), 10 mM 4-(2-hydroxyethyl)-1-piperazineethanesulfonic acid (HEPES, Sigma-Aldrich, Taufkirchen, Germany) and 2 mM L-Glutamine (Sigma) at 37 °C with 5% CO_2_ and humidified atmosphere. Lpd WT MEF and Lpd KO MEF [21] were cultured in DMEM (Thermo Fisher Scientific), 10% FBS (PAN-Biotech) and 2 mM L-Glutamine (Sigma) at 37 °C with 8.5% CO_2_ and humidified atmosphere. All cell lines were authenticated using short-tandem repeat DNA profiling and tested as mycoplasma negative.

### 4.2. Small Interfering RNA (siRNA) and Transfection

Transfections of EGFR siRNA (5′-GGCACGAGUAACAAGCUCAtt-3′), FOS siRNA (5′-CCTGCTGAAGGAGAAGGAAtt-3′), Lpd siRNA #1 (5′-GAGAUUCCAGCAGUUUUCUtt-3′), Lpd siRNA #2 (5′-GGAUCCGCAUUGCAAAGUAtt-3′), RICTOR siRNA (5′-GCAGUAAAUUAUAUGCCAAUUtt-3′), SRC siRNA (5′-GGCUGAGGAGUGGUAUUUUtt-3′) and non-specific siRNA as control (5′-GCAGCUAUAUGAAUGUUGUtt-3′) were purchased from Eurofins MWG. PRKAA2 siRNA (5′-GGUGAAUUAUUUGACUACAtt-3′), EIF2A siRNA (5′-CCACCACAUUUUACAGAAAtt -3′), NFKBIA siRNA (5′-GAGCUCCGAGACUUUCGAGGAAAUA-3′), MKK6 siRNA (5′-GGCUUGCAUUUCUAUUGGAtt-3′), RSK2 siRNA (5′-GGACAUGAAAAGGCAGAUCtt-3′), TAK1 siRNA (5′-GCAACAGAGUGAAUCUGGACGUUUA-3′) were obtained from Thermo Fisher Scientific. siRNA transfection with Oligofectamine (Invitrogen, Waltham, MA, USA) was performed as published [9].

### 4.3. Lamellipodin Plasmid and Transfection

Transient transfection of mCherry and mCherry-tagged Lamellipodin plasmids [19] using Lipofectamine LTX (Invitrogen) was carried out according to the manufacturer’s instructions and as published [9].

### 4.4. 3D Spheroid Invasion Assay

Glioblastoma spheroids were generated by seeding 10,000 cells in 96-well plates coated with 1% agarose. After 2–3 days spheroids were embedded in a 1 mg/mL 3D collagen type I matrix (Corning, Corning, NY, USA). Invasion was imaged 24 h to 72 h with Axiovert 200 (Carl Zeiss Microscopy GmbH, Jena, Germany) and distance of invading cells was measured as indicated [8].

### 4.5. Colony Formation Assay

Clonogenicity was determined by plating single cells (A172, DD-HT7606, DD-T4, Lpd WT MEF, Lpd KO MEF, LN229, LN405, U87MG, U138MG, U251MG and U343MG) in collagen type I coated (1 μg/cm^2^, BD Biosciences) 6-well culture plates as published [8]. Cell colonies were fixed with 80% Ethanol and stained with Coomassie blue (Merck Millipore, Burlington, MA, USA) after cell line dependent incubation time. The incubation times were as follows: A172 12 days, DD-HT7606 10 days, DD-T4 10 days, Lpd MEF 7 days, LN229 7 days, LN405 11 days, U87MG 10 days, U138MG 15 days, U251MG 10 days and U343MG 10 days. Cell colonies with more than 50 cells were manually counted by using the stereo microscope Stemi 2000 (Carl Zeiss Microscopy GmbH). For calculation of normalized surviving fraction, the surviving fractions of indicated knockdown cells were divided by corresponding surviving fractions of control knockdown cells. The Synergism Assessment Factor (S) was calculated as described in [68].

### 4.6. Radiation Exposure

X-ray irradiation was delivered under room temperature using single doses of 200 kV X-rays (Yxlon Y.TU 320; Yxlon, Hamburg, Germany) filtered with 0.5 mm Cu. The approximated dose-rate was 1.3 Gy/min at 20 mA. The absorbed dose was measured using a Duplex dosimeter (PTW, Freiburg, Germany).

### 4.7. Proliferation Assay

Cell proliferation was assayed by seeding 1000 cells in collagen type I coated (1 μg/cm^2^, BD Biosciences) 24-well culture plates. For cell counting, cells were trypsinized, diluted in cell culture medium and counted using a Neubauer chamber up to 5 days after seeding. Doubling time (D) was calculated comparing day 0 (*n*_d0_) to counted cells at day 3 to 5 (*n*_dn_) by using the following formula (1):

D = (*T*_0_ − *T*_n_)/log2(*n*_dn_ − *n*_d0_) (1)

### 4.8. Total Protein Extracts and Western Blotting

Cells were lysed with GST lysis buffer (50 mM Tris HCl (AppliChem, Darmstadt, Germany), 200 mM NaCl (Roth, Karlsruhe, Germany), 1% NP-40 (Sigma-Aldrich, Taufkirchen, Germany), 2 mM MgCl_2_ (Merck, Kenilworth, NJ, USA), 10% glycerol (Sigma), pH 7.4, substituted with 1 mM Na3VO4 (Sigma), 10 mM NaF (Roth) and Complete™ Protease Inhibitor Cocktail (Roche, Basel, Switzerland) [21]). Measurement of protein concentrations, SDS-PAGE and Western blotting were performed as previously described in [8]. Antibodies were purchased as follows: β-actin (#A5441, Sigma), Caspase 3 (#9662, Cell Signaling Technology, Frankfurt a. M., Germany), EGFR (#2232, Cell Signaling), EGFR pY1068 (#44-788G, Invitrogen, Waltham, MA, USA), EGFR pY1173 (#44-794G, Invitrogen), Lamellipodin pab 3917 [20], Lamellipodin p-Y426 [16], mCherry (#ab183628, Abcam, Cambridge, UK), Lamellipodin (#91138, Cell Signaling), RICTOR (#9964, Cell Signaling), RICTOR T1135 (#3806, Cell Signaling) HRP-conjugated anti-rabbit secondary antibody (NXA931, GE Healthcare, Chicago, IL, USA) and HRP-conjugated anti-mouse secondary antibody (NA934V, GE Healthcare). ECL Prime Western Blotting Detection Reagent (GE Healthcare) was used for detection of proteins on X-ray films (GE Healthcare) for Figure 1D, Figure 2A, Figure 5E and Figure S2F and Fusion FX (Vilber Lourmat GmbH, Eberhardzell, Germany) for Figure 1A,G, Figure 2D,G, Figure 4A,C, Figure 6A,F,H, Figures S5 and S6D and Figure 7A,D. In Figures S9–S18, the uncropped Western blot images are shown. All protein bands were normalized to the corresponding β-actin. Phosphorylated proteins were further normalized to total protein by dividing the intensity of the normalized phosphorylated protein by the intensity of the normalized total protein. The resulting values are relative to siC 0 Gy treated cells (fold change).

### 4.9. Immunoprecipitation and Mass Spectrometry Analysis

A172 cells were cultured on collagen type I coated 150 mm culture dishes (1 μg/cm^2^, BD Biosciences) for 24 h, following irradiation with 6 Gy X-rays. Cells were lysed 1 h post 6 Gy X-ray irradiation with GST Lysis buffer. BCA assay (Thermo Fisher Scientific) was used to determine protein concentration. For each condition, 1000 µg cell lysate was pre-cleared using 100 μL of a protein A/G Sepharose beads slurry (50% v/v, Biotrend, Cologne, Germany). Lamellipodin (#91138, Cell Signaling) or IgG from rabbit serum (negative control, Sigma) primary antibodies together with 100 µL of protein A/G Sepharose beads (Biotrend) were added to protein lysate and rotated overnight at 4 °C. Immunoprecipitates were washed three times with cold GST lysis buffer. Subsequently, immunoprecipitates were boiled in 50 µL sample buffer and subjected to SDS-PAGE and Western blot analysis. HRP-conjugated anti-rabbit secondary antibody (GTX221666-01, GeneTex, Irvine, CA, USA) was used for detection. Remaining immunoprecipitates were transferred to the Genomics and Proteomics Core Facility (German Cancer Research Center, Heidelberg, Germany) for mass-spectrometry-based protein analysis on an Orbitrap-based mass spectrometer Q-Exactive-HF-X (Thermo Fisher Scientific). Analysis was achieved by the label-free quantification approach based on the MaxLFQ algorithm [69]. Proteins were selected by analyzing the ratio of label-free quantification values of Lpd and IgG immunoprecipitation and number of peptides of Lpd and IgG immunoprecipitation. Proteins with ratios of at least two were selected and analyzed using Reactome [70] and KEGG Pathway Database [71].

For immunoprecipitation upon pharmacological inhibition of EGFR, U343MG were cultured on collagen type I coated 150 mm culture dishes (1 μg/cm^2^, BD Biosciences) for 24 h and then treated with 10 µM Tarceva^®^ (Roche) or 10 µM dimethylsulfoxid (DMSO) 1 h prior to X-ray irradiation.

### 4.10. Proximity Ligation Assay

The proximity ligation Duolink In Situ Red Starter Kit Mouse/Rabbit (Sigma Aldrich, St. Louis, MO, USA) was performed according to the manufacturer’s protocol and as described in [72]. Cells were incubated with Lamellipodin pab 3917 [20] and RICTOR (#MA5-15681, Invitrogen). Samples were analyzed with AxioImager M1 microscope (Carl Zeiss Microscopy) with a magnification of 20×.

### 4.11. Immunofluorescence Staining and Microscopy

To analyze localization of Lpd, glioblastoma cells were stained with *Lamellipodin* pab 3917, AlexaFluor488anti-rabbit (A32731, Life Technologies, Carlsbad, CA, USA), AlexaFluor633 Phalloidin (A22284, Life Technologies) and mounted using ProLong Diamant Antifade Mountant with DAPI (Thermo Fisher Scientific) as described [9]. Images were acquired using LSM980 Airyscan 2 (Carl Zeiss Microscopy).

### 4.12. Phosphoarray Analysis

Following siRNA transfection, A172 cells were cultured on collagen type I coated 100 mm culture dishes (1 μg/cm^2^, BD Biosciences) for 24 h and irradiated with 6 Gy X-rays 48 h post incubation. Cells were harvested 1 h after irradiation and whole-cell lysates were prepared. The Human Phosphorylation Pathway Profiling Array C55 (RayBiotech Life Inc., Peachtree Corners, GA, USA) was performed according to the manufacturer’s instructions using 150 µg protein per condition. Phosphosite changes of at least 30% reduction or 50% increase were selected and classified in signaling pathways using Reactome [70] and KEGG Pathway Database [71]. The network of molecular interaction with Lpd and RICTOR was generated with Cytoscape [73].

### 4.13. Statistical Analysis

Mean +/− standard deviation (SD) was calculated from at least three independent biological experiments (indicated as *n*). Statistical evaluation was determined with two-sided Student’s *t*-test using Microsoft Excel 2016. A *p*-value of less than 0.05 was considered statistically significant.

## 5. Conclusions

Altogether, our results highlight the multifaceted functions of the cytoskeleton regulating protein Lamellipodin in invasion, proliferation and radiosensitivity of glioblastoma cells. At the molecular level, we discovered a so-far-unknown protein complex consisting of Lamellipodin and RICTOR and that Lamellipodin mediates glioblastoma radiation survival via EGFR signaling. Thus, the involvement of cytoskeleton regulators such as Lamellipodin to the cellular response to X-rays adds an additional facet to the complex glioblastoma therapy resistance network.

## Data Availability

The data presented in this study are available in this article (and supplementary material).

## Supplementary Materials

The following are available online at https://www.mdpi.com/article/10.3390/cancers13215337/s1: Figure S1: Lamellipodin promotes glioblastoma invasiveness; Figure S2: Lamellipodin promotes radiation survival and proliferation in glioblastoma cells; Figure S3: Lamellipodin and EGFR-MAPK signaling proteins co-regulate glioblastoma radiosensitivity; Figure S4: Lamellipodin and EGFR-MAPK signaling proteins co-regulate glioblastoma radiosensitivity; Figure S5: EGFR and Lpd expression and phosphorylation status in glioblastoma cells; Figure S6: Lamellipodin and RICTOR interact; Figure S7: Lamellipodin and RICTOR co-control glioblastoma radiosensitivity and invasion; Figure S8: Lamellipodin and RICTOR jointly regulate radiosensitivity via EGFR; Figures S9 and S10: Compilation of uncropped Western blots for Figure 1; Figures S11 and S12: Compilation of uncropped Western blots for Figure 2; Figure S13: Compilation of uncropped Western blots for Figure S2; Figures S14 and S15: Compilation of uncropped Western blots for Figure 4 and Figure S5; Figure S16: Compilation of uncropped Western blots for Figure 5 and Figure S6; Figure S17: Compilation of uncropped Western blots for Figure 6 and Figure S7; Figure S18: Compilation of uncropped Western blots for Figure 6F,H; Table S1: Synergistic and additive effects of siLpd knockdown and 6 Gy X-ray irradiation; Table S2: Mean intensity of indicated phosphorylation sites at 0 Gy and 6 Gy upon siC and siLpd#1 treatment; Table S3: Fold change normalized to 0 Gy of indicated phosphorylation sites calculated from mean intensity; Table S4: Lamellipodin-bound proteins detected by mass spectrometry following Lamellipodin immunoprecipitation from unirradiated cells; Table S5: Lamellipodin bound proteins detected by mass spectrometry following Lamellipodin immunoprecipitation from 6-Gy irradiated cells.
