# Supplementary material for: Lamellipodin-RICTOR Signaling Mediates Glioblastoma Cell Invasion and Radiosensitivity Downstream of EGFR
Source: Cancers (Basel). 2021 Oct 24;13(21):5337. doi: 10.3390/cancers13215337 (PMC8582497; doi:10.3390/cancers13215337)
Supplement: Supplementary file 1 [file cancers-13-05337-s001.zip › cancers-1386069 supplementary/Cancers_Moritz et al_Original Western Blots_20211020.pdf]

cancers

Original Western Blots

Stefanie Moritz [1], Matthias Krause [2], Jessica Schlatter [2], Nils Cordes [1,3,4,5], Anne Vehlow [1,5,6,*]

[1] OncoRay – National Center for Radiation Research in Oncology, Faculty of Medicine, Technische Universität Dresden, Fetscherstr. 74, PF 41, 01307 Dresden, Germany; Stefanie.Moritz@uniklinikum-dresden.de (S.M.); Nils.Cordes@OncoRay.de (N.C.)
[2] Randall Centre of Cell and Molecular Biophysics, New Hunt's House, Guy's Campus, King's College London, London, SE1 1UL, UK; matthias.krause@kcl.ac.uk (M.K.); jessica.schlatter@kcl.ac.uk (J.S.)
[3] Institute of Radiooncology – OncoRay, Helmholtz-Zentrum Dresden - Rossendorf, Dresden, Germany, Bautzner Landstr. 400, 01328 Dresden, Germany
[4] Department of Radiotherapy and Radiation Oncology, University Hospital Carl Gustav Carus, Technische Universität Dresden, Dresden, Germany, Fetscherstr. 74, PF 50, 01307 Dresden, Germany
[5] German Cancer Consortium (DKTK), Partner Site Dresden, and German Cancer Research Center (DKFZ), Heidelberg, Germany, Im Neuenheimer Feld 280, 69192 Heidelberg, Germany
[6] National Center for Tumor Diseases (NCT), Partner Site Dresden, Germany: German Cancer Research Center (DKFZ), Heidelberg, Germany, Im Neuenheimer Feld 280, 69192 Heidelberg, Germany
* Correspondence: Anne.Vehlow@OncoRay.de; Tel.: +49 (0)351–458–7403; Fax: +49 (0)351–458–7311 (A.V.)

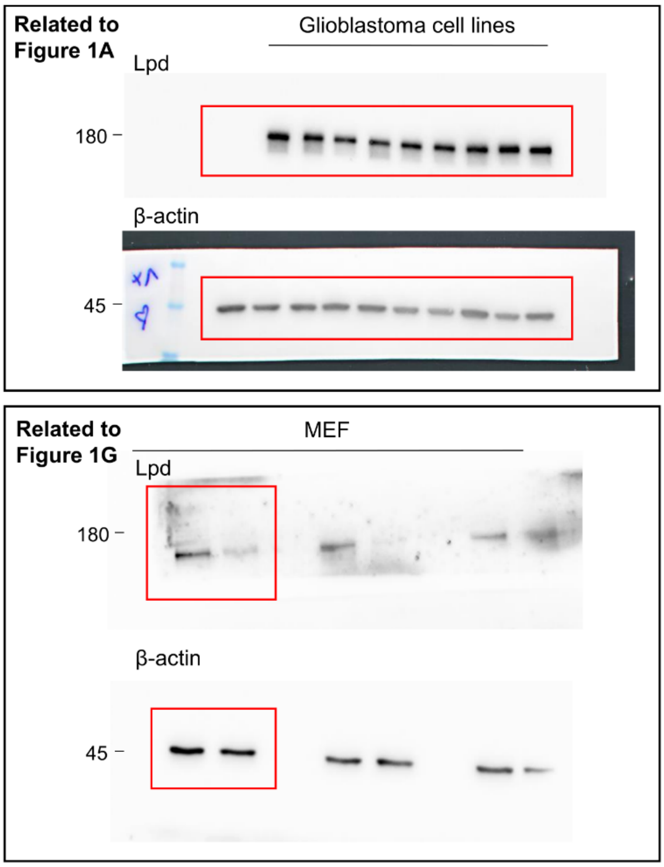

Figure S9. Compilation of uncropped western blots for Figure 1A,G. The corresponding main figures where the cropped versions are shown are specified on top of the blots. The red rectangles display the areas of the plots that were used in Figure 1A and G. The molecular weight standard [kDa] per blot is located on the left. The detected protein is indicated on top of the blots.

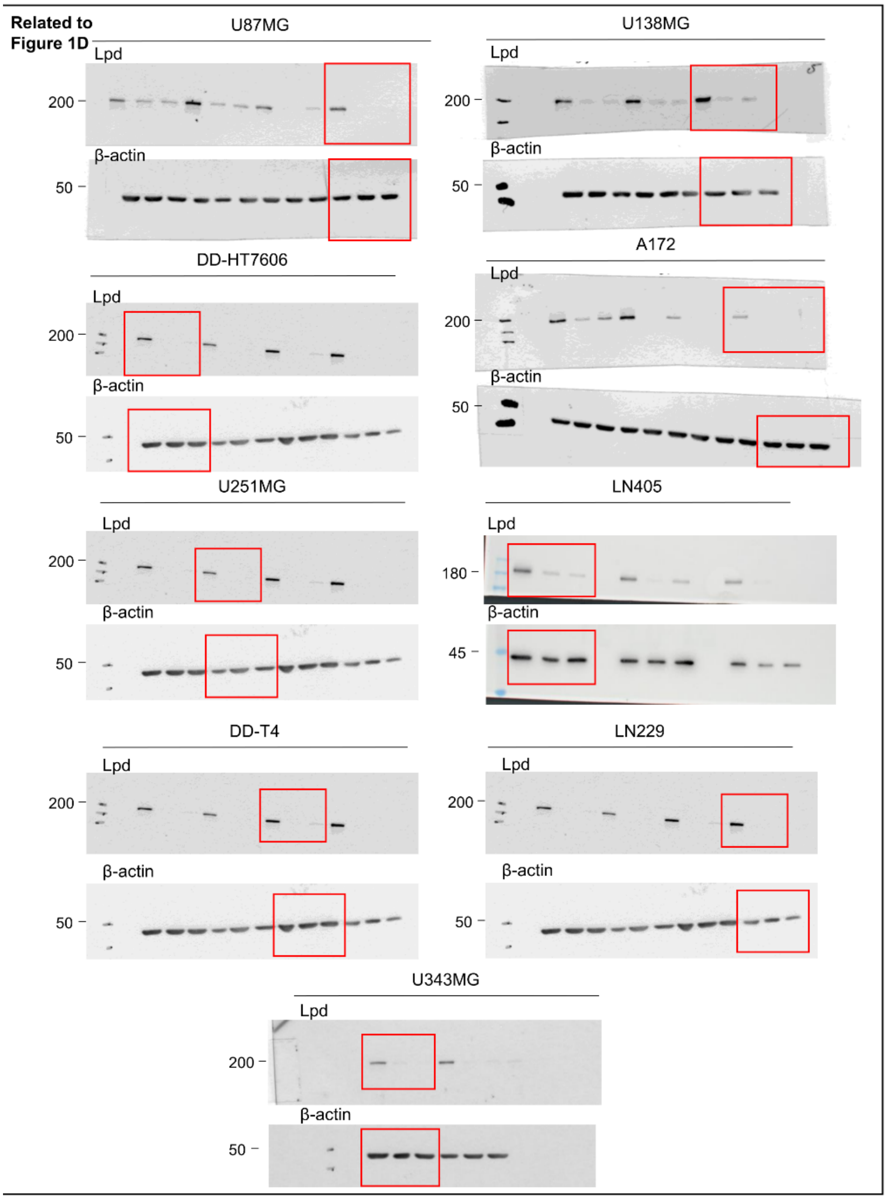

Figure S10. Compilation of uncropped western blots for Figure 1D. The corresponding main figures where the cropped versions are shown are specified on top of the blots. The red rectangles display the areas of the plots that were used in Figure 1D. The molecular weight standard [kDa] per blot is located on the left. The detected protein is indicated on top of the blots.

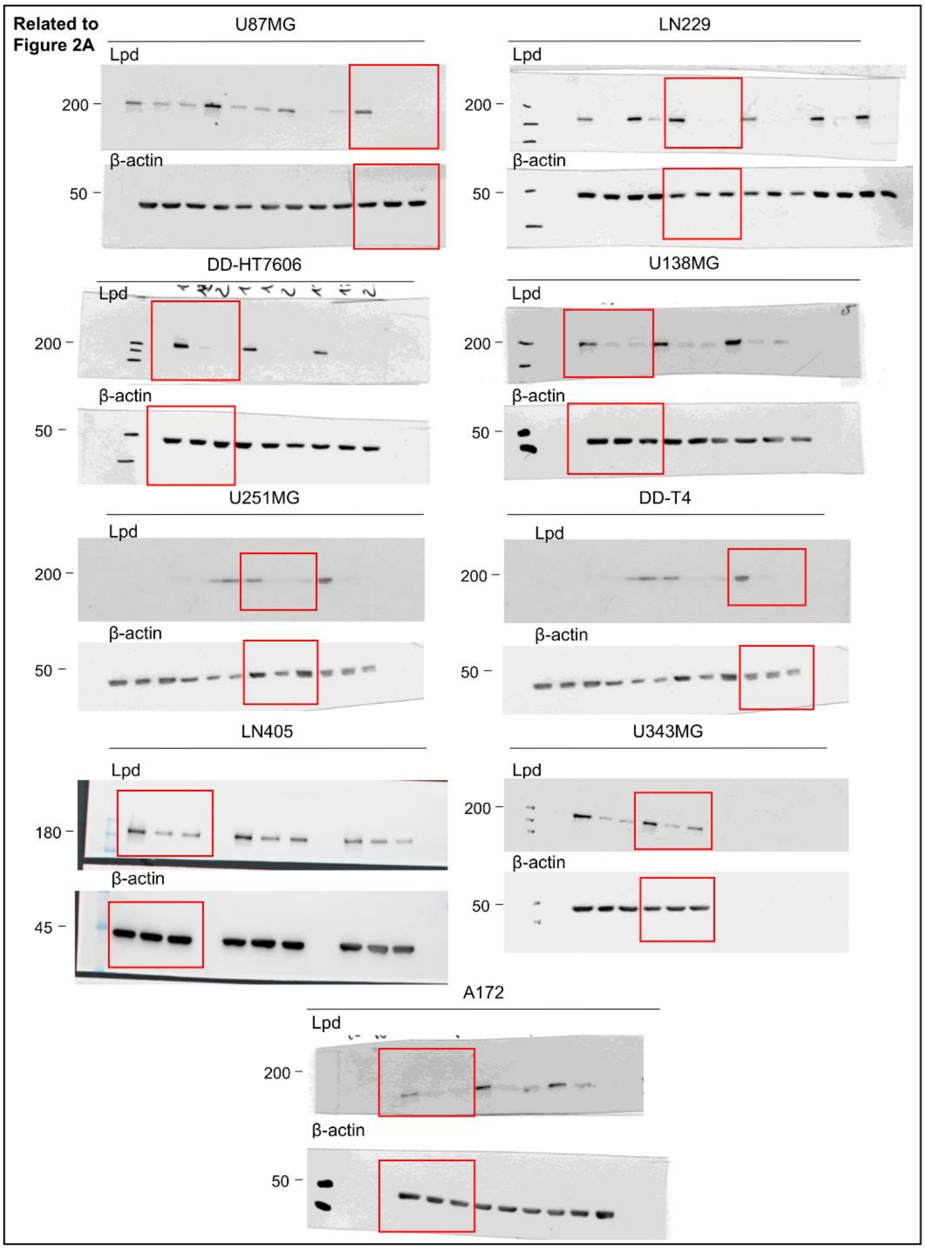

Figure S11. Compilation of uncropped western blots for Figure 2A. The corresponding main figures where the cropped versions are shown are specified on top of the blots. The red rectangles display the areas of the plots that were used in Figure 2A. The molecular weight standard [kDa] per blot is located on the left. The detected protein is indicated on top of the blots.

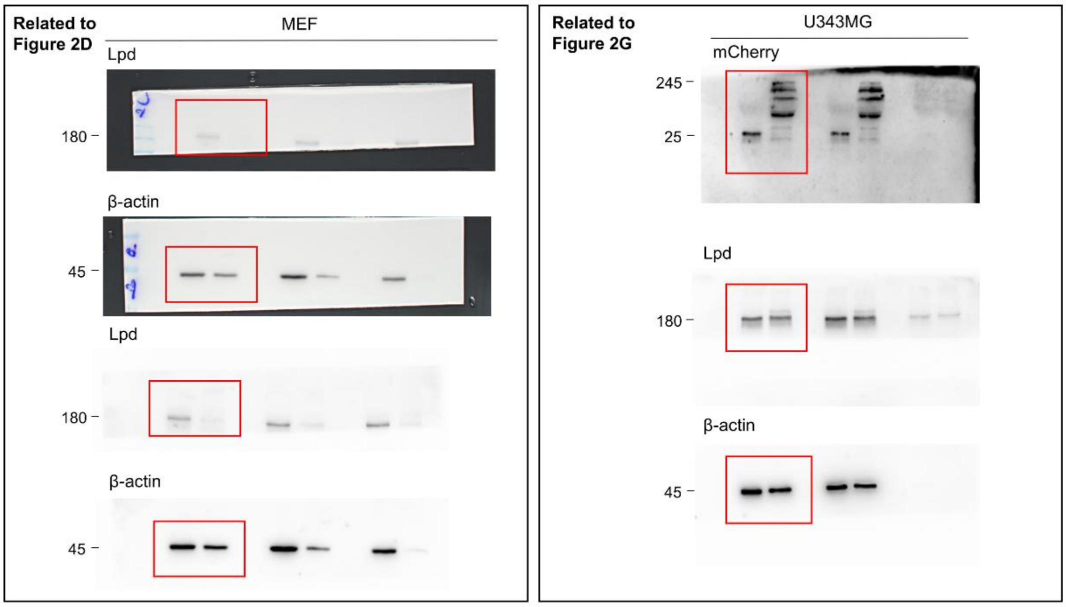

Figure S12. Compilation of uncropped western blots for Figure 2D,G. The corresponding main figures where the cropped versions are shown are specified on top of the blots. The red rectangles display the areas of the plots that were used in Figure 2D,G. The molecular weight standard [kDa] per blot is located on the left. The detected protein is indicated on top of the blots.

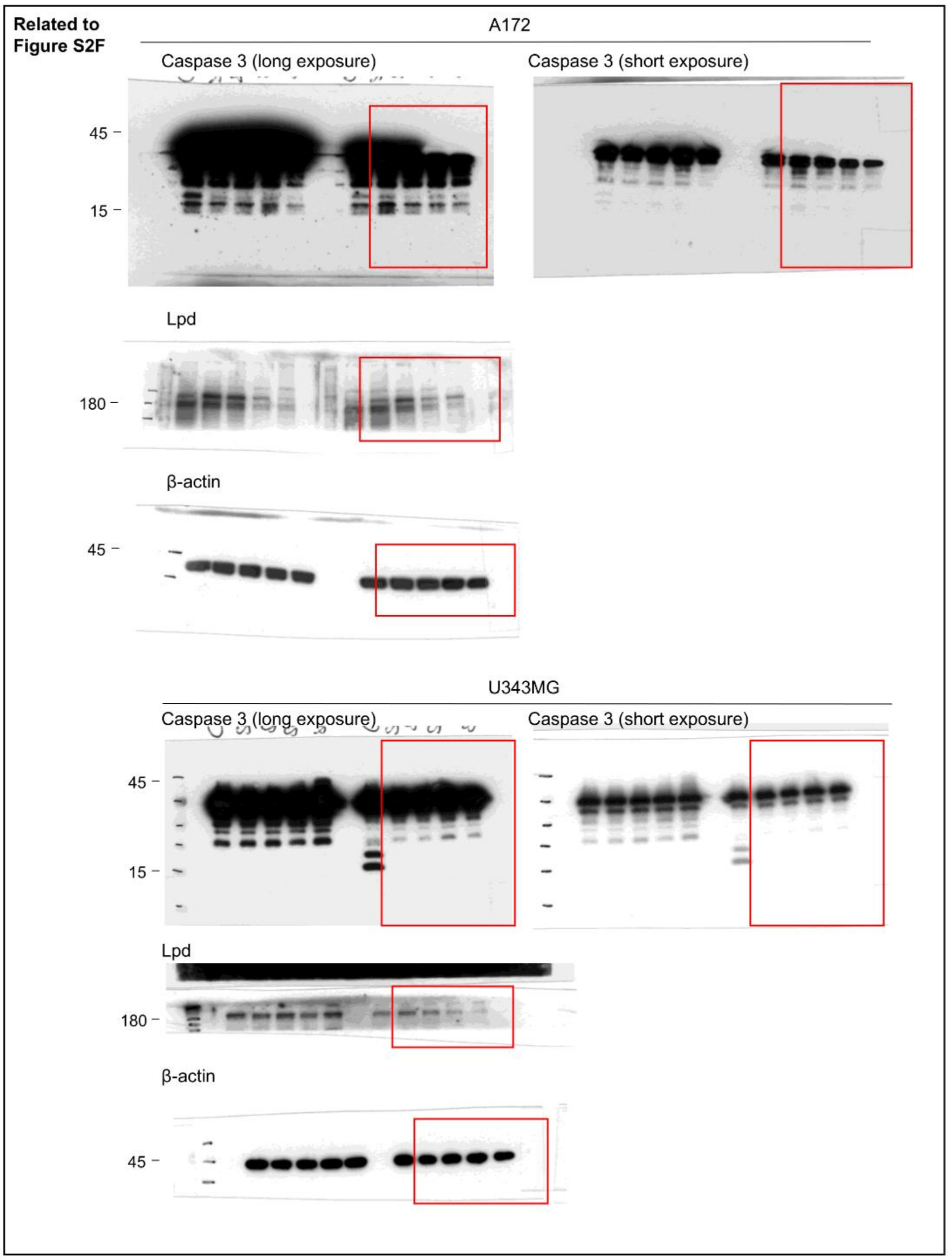

Figure S13. Compilation of uncropped western blots for Figure S2. The corresponding main figures where the cropped versions are shown are specified on top of the blots. The red rectangles display the areas of the plots that were used in Figure S2. The molecular weight standard [kDa] per blot is located on the left. The detected protein is indicated on top of the blots.

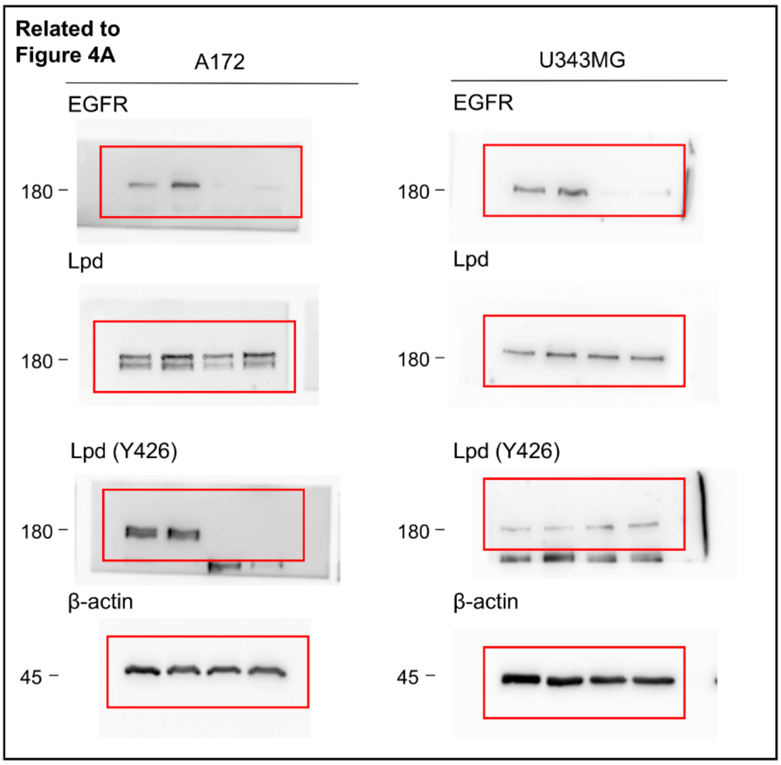

Figure S14. Compilation of uncropped western blots for Figure 4A. The corresponding main figures where the cropped versions are shown are specified on top of the blots. The red rectangles display the areas of the plots that were used in Figure 4A. The molecular weight standard [kDa] per blot is located on the left. The detected protein is indicated on top of the blots.

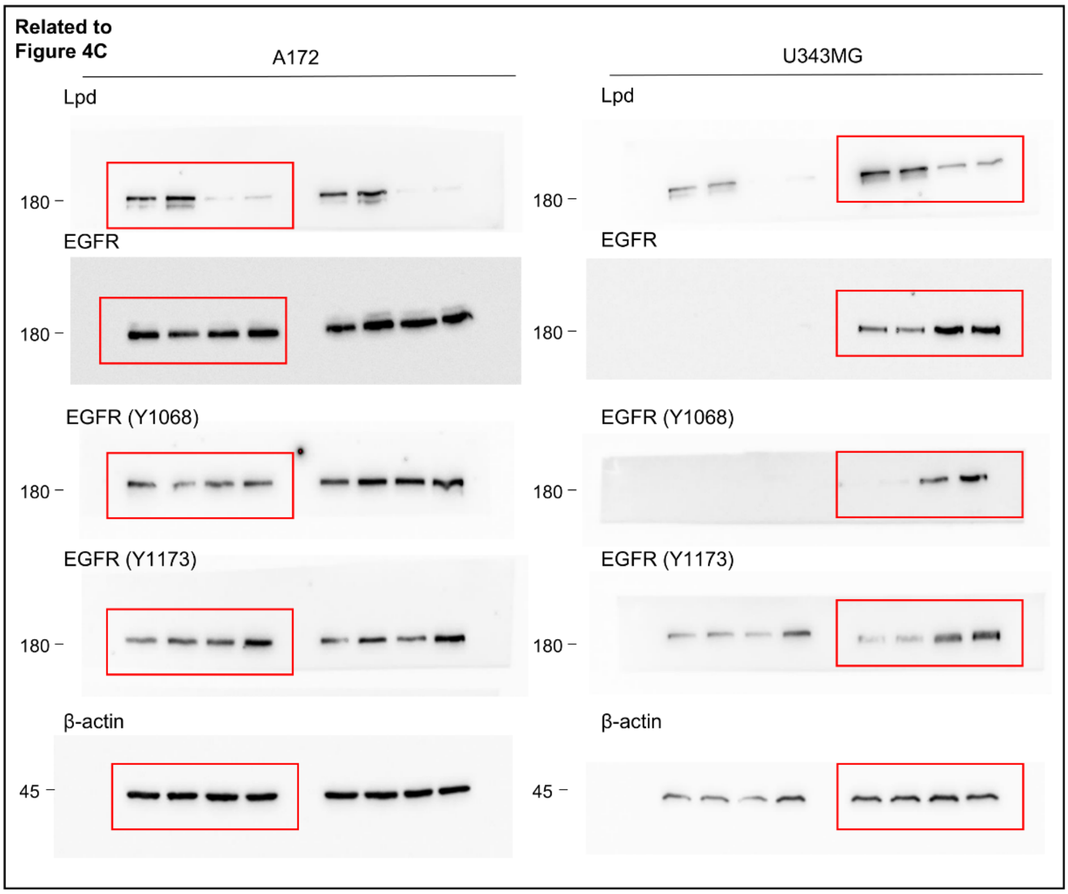

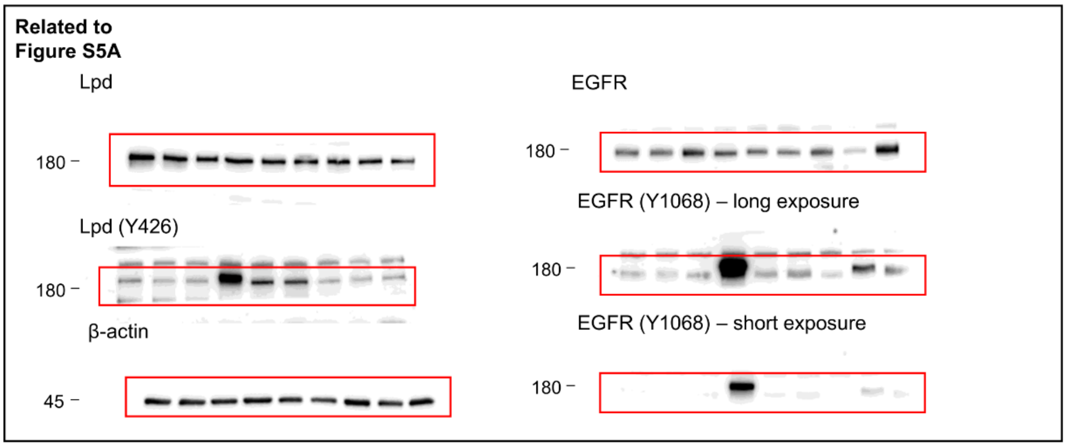

Figure S15. Compilation of uncropped western blots for Figure 4C and S5. The corresponding main figures where the cropped versions are shown are specified on top of the blots. The red rectangles display the areas of the plots that were used in Figure 4C and Figure S5. The molecular weight standard [kDa] per blot is located on the left. The detected protein is indicated on top of the blots.

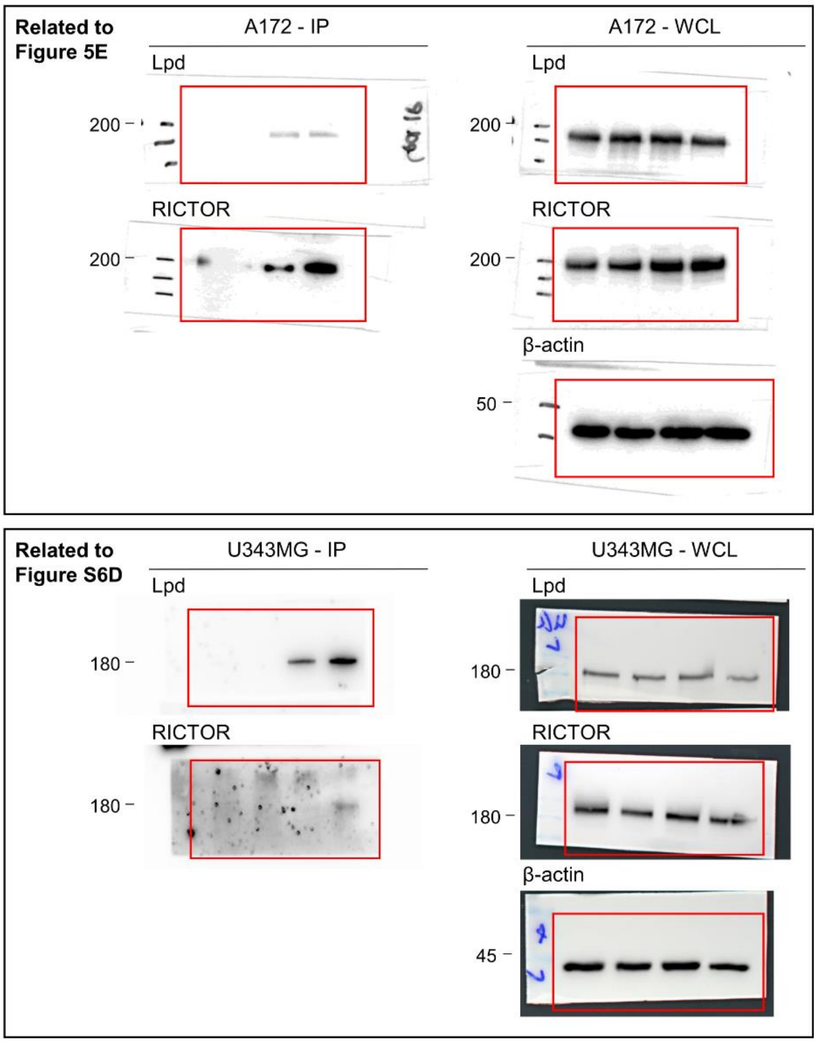

Figure S16. Compilation of uncropped western blots for Figure 5 and S6. The corresponding main figures where the cropped versions are shown are specified on top of the blots. The red rectangles display the areas of the plots that were used in Figure 5 and S6. The molecular weight standard [kDa] per blot is located on the left. The detected protein is indicated on top of the blots.

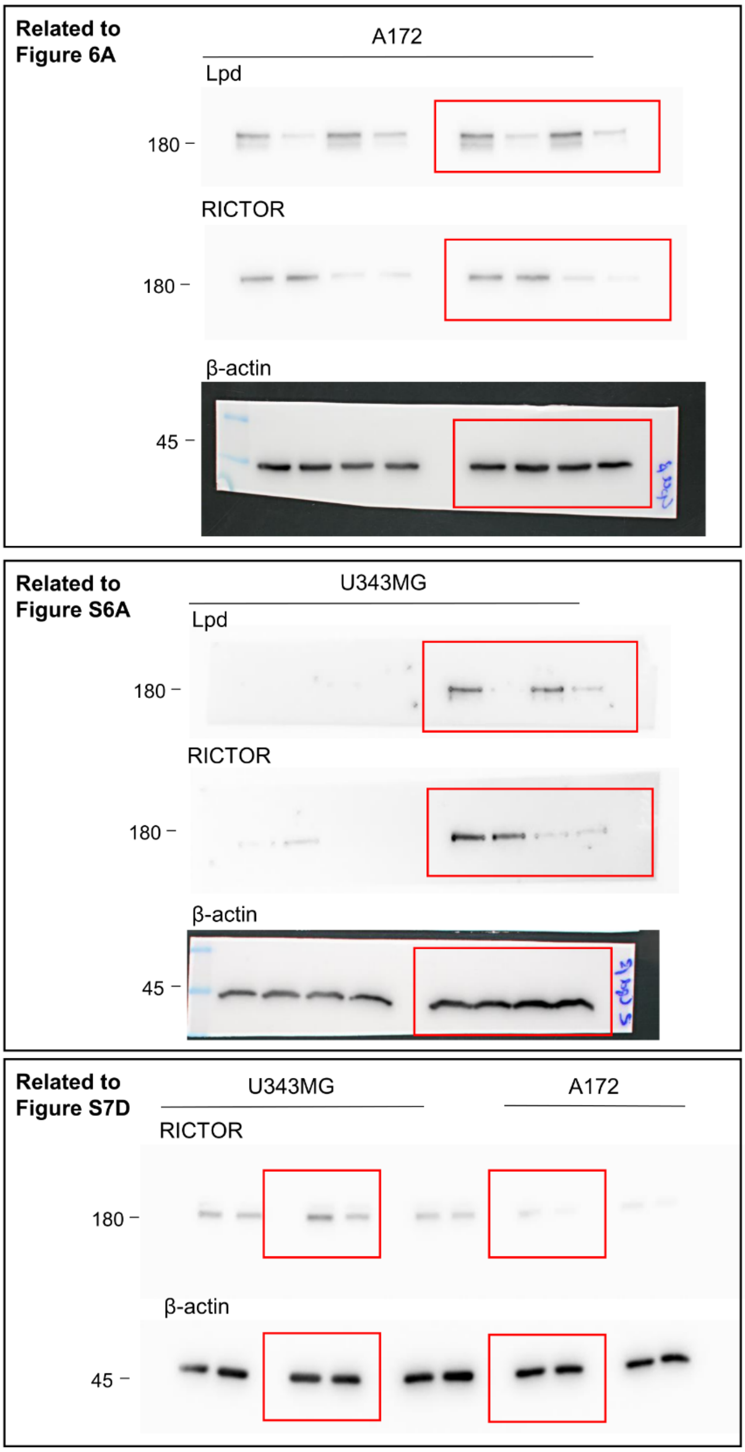

Figure S17. Compilation of uncropped western blots for Figure 6A and S7. The corresponding main figures where the cropped versions are shown are specified on top of the blots. The red rectangles display the areas of the plots that were used in Figure 6A and S7. The molecular weight standard [kDa] per blot is located on the left. The detected protein is indicated on top of the blots.

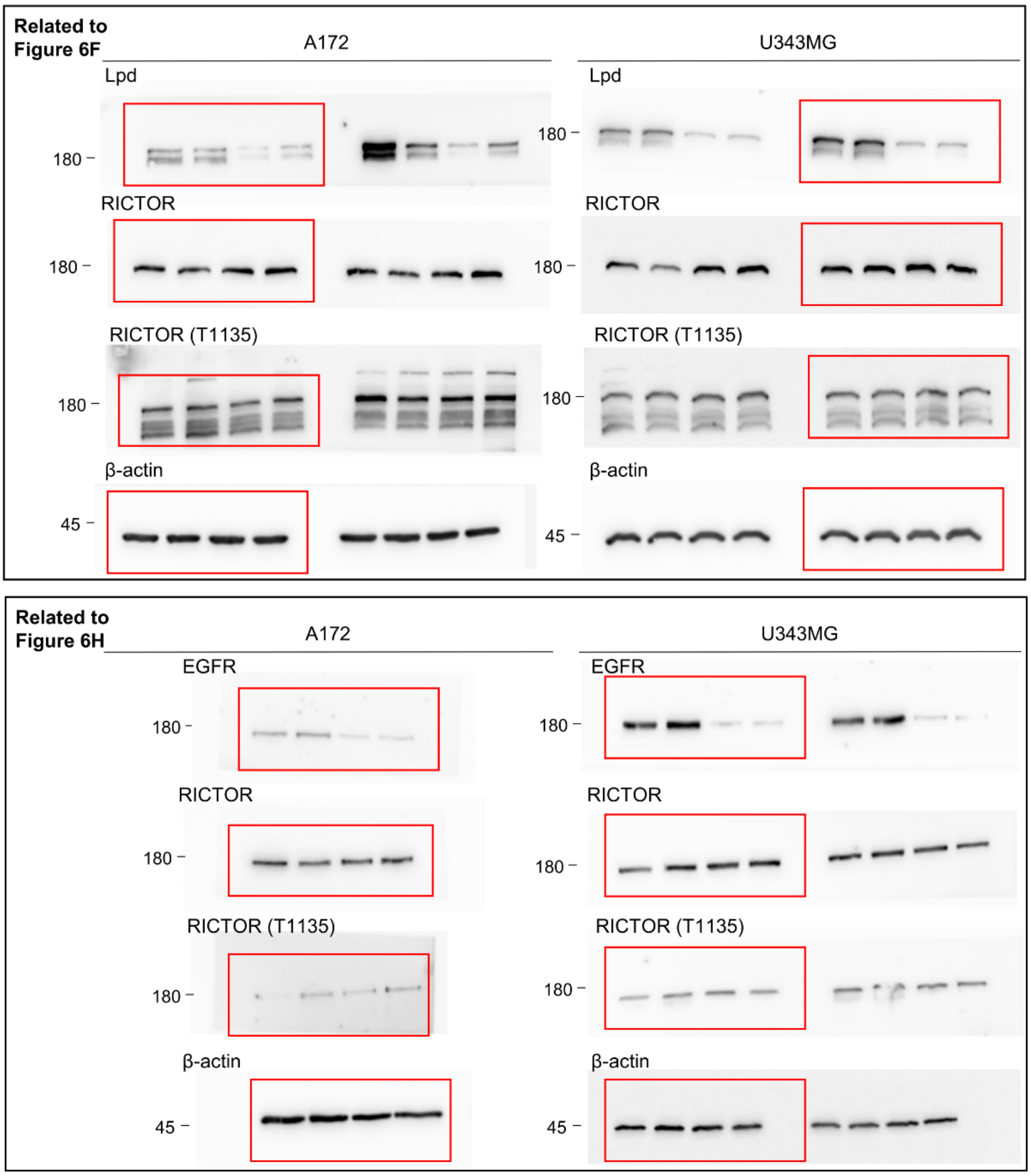

Figure S18. Compilation of uncropped western blots for Figure 6F,H. The corresponding main figures where the cropped versions are shown are specified on top of the blots. The red rectangles display the areas of the plots that were used in Figure 6F,H. The molecular weight standard [kDa] per blot is located on the left. The detected protein is indicated on top of the blots.
