# Supplementary material for: Lamellipodin-RICTOR Signaling Mediates Glioblastoma Cell Invasion and Radiosensitivity Downstream of EGFR
Source: Cancers (Basel). 2021 Oct 24;13(21):5337. doi: 10.3390/cancers13215337 (PMC8582497; doi:10.3390/cancers13215337)
Supplement: Supplementary file 1 [file cancers-13-05337-s001.zip › cancers-1386069 supplementary/Cancers_Moritz et al_Supplementary Material_20211020.pdf]

cancers

Supplementary Materials: Lamellipodin-RICTOR signaling mediates glioblastoma cell invasion and radiosensitivity downstream of EGFR

Stefanie Moritz [1], Matthias Krause [2], Jessica Schlatter [2], Nils Cordes [1,3,4,5], Anne Vehlow [1,5,6,*]

1 OncoRay – National Center for Radiation Research in Oncology, Faculty of Medicine, Technische Universität Dresden, Fetscherstr. 74, PF 41, 01307 Dresden, Germany; Stefanie.Moritz@uniklinikum-dresden.de (S.M.); Nils.Cordes@OncoRay.de (N.C.)
2 Randall Centre of Cell and Molecular Biophysics, New Hunt's House, Guy's Campus, King's College London, London, SE1 1UL, UK; matthias.krause@kcl.ac.uk (M.K.); jessica.schlatter@kcl.ac.uk (J.S.)
3 Institute of Radiooncology – OncoRay, Helmholtz-Zentrum Dresden - Rossendorf, Dresden, Germany, Bautzner Landstr. 400, 01328 Dresden, Germany
4 Department of Radiotherapy and Radiation Oncology, University Hospital Carl Gustav Carus, Technische Universität Dresden, Dresden, Germany, Fetscherstr. 74, PF 50, 01307 Dresden, Germany
5 German Cancer Consortium (DKTK), Partner Site Dresden, and German Cancer Research Center (DKFZ), Heidelberg, Germany, Im Neuenheimer Feld 280, 69192 Heidelberg, Germany
6 National Center for Tumor Diseases (NCT), Partner Site Dresden, Germany: German Cancer Research Center (DKFZ), Heidelberg, Germany, Im Neuenheimer Feld 280, 69192 Heidelberg, Germany
* Correspondence: Anne.Vehlow@OncoRay.de; Tel.: +49 (0)351–458–7403; Fax: +49 (0)351–458–7311 (A.V.)

Supplementary Figures

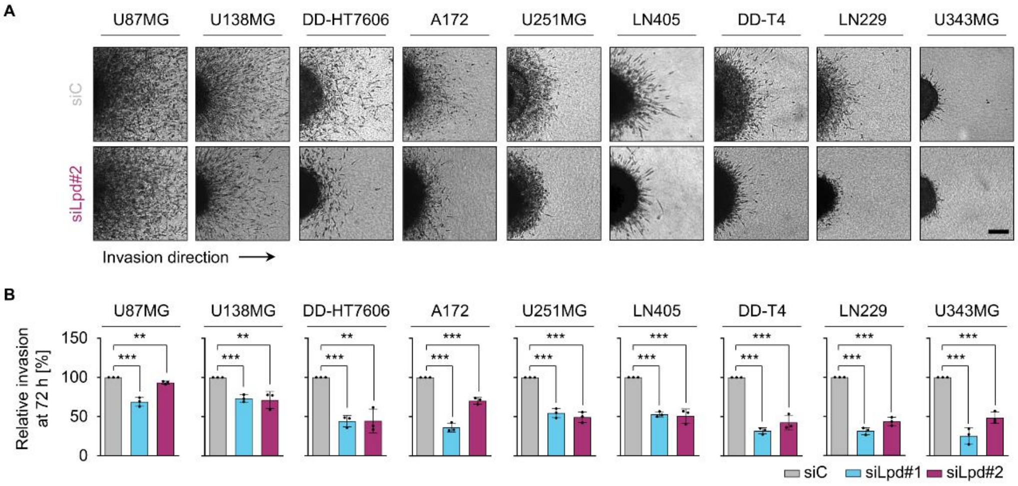

Figure S1. Lamellipodin promotes glioblastoma invasiveness. (**A**) Representative phase contrast images of indicated glioblastoma spheroids invading in 3D type I collagen type matrix upon Lpd or control knockdown (t = 72 h). Scale bar: 200 µm. (**B**) Relative invasion distance of indicated glioblastoma cell lines upon control and Lpd-specific siRNA knockdown in 3D type I collagen recorded after 72 h. siRNA, small interfering RNA; C, control; Lpd, Lamellipodin.

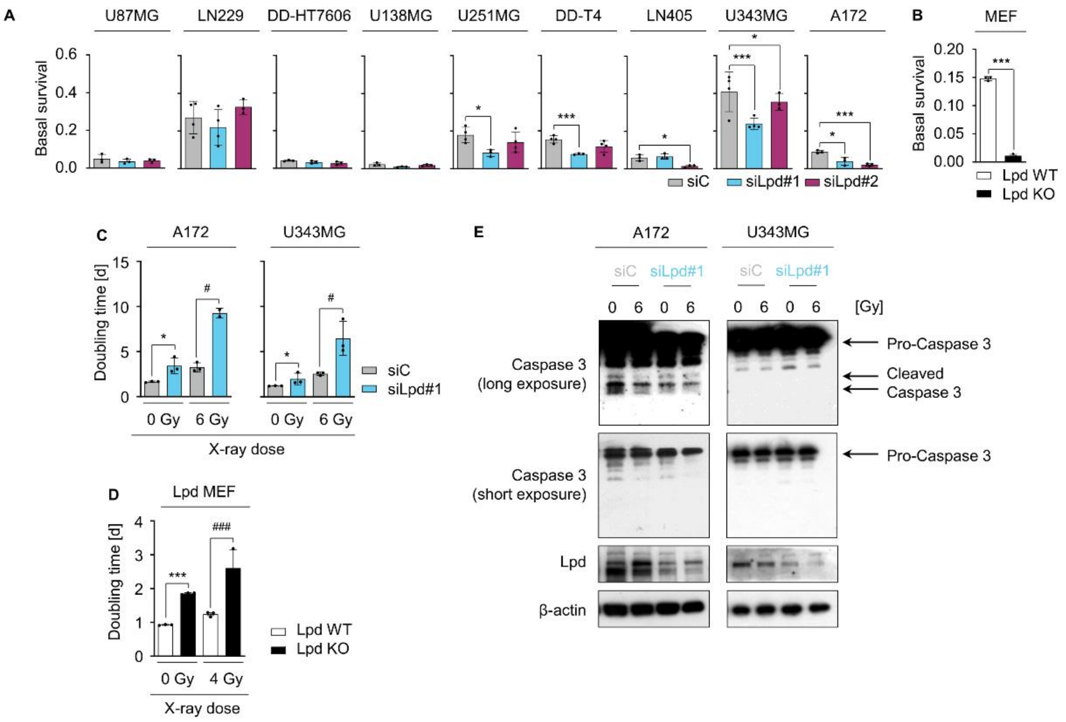

Figure S2. Lamellipodin promotes radiation survival and proliferation in glioblastoma cells. (**A**) Basal survival of Lpd and control depleted, unirradiated glioblastoma cells as well as (**B**) Lpd WT MEF and Lpd KO MEF. (**C**) Doubling time of A172 and U343MG cells after siRNA-mediated knockdown of Lpd and control after 6 Gy irradiation and (**D**) in Lpd WT MEF and Lpd KO MEF upon 4 Gy irradiation. Doubling time was calculated by comparing cell counts of day 3-5 to day 0. All results are presented as mean ± SD (n = 3-4, two-sided Student's t-test, *p < 0.05, **p < 0.01, ***p < 0.001). (E) Representative Western blot of Caspase 3, Lpd and β-actin of control and Lpd silenced A172 and U343MG (n = 3) 24 h after irradiation. siRNA, small interfering RNA; C, control; Lpd, Lamellipodin; MEF, mouse embryonic fibroblasts; WT, wild type; KO, knockout.

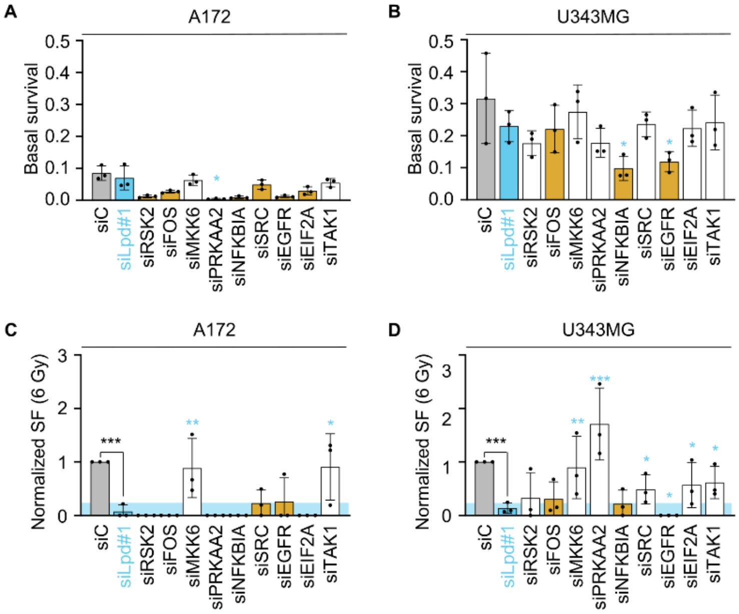

Figure S3. Lamellipodin and EGFR-MAPK signaling proteins co-regulate glioblastoma radiosensitivity. (**A**) Basal survival (0 Gy) upon siRNA mediated knockdown of Lpd and the identified proteins in A172 and (**B**) U343MG. (**C**) Normalized clonogenic radiation survival (6 Gy) of A172 cells and (**D**) U343MG cells upon siRNA mediated knockdown of Lpd and the identified proteins. Yellow columns indicate a surviving fraction comparable to single Lpd knockdown, whereas white columns indicate a differing survival. The blue area represents the normalized surviving fraction after Lpd knockdown and 6 Gy irradiation. Results present mean ± SD (n = 3, two-sided Student's t-test, n.s., not significant, siLpd / siC ***p < 0.001, siRNA / siLpd *p < 0.05, **p < 0.01, ***p < 0.001). siRNA, small interfering RNA; C, control; Lpd, Lamellipodin; SF, surviving fraction.

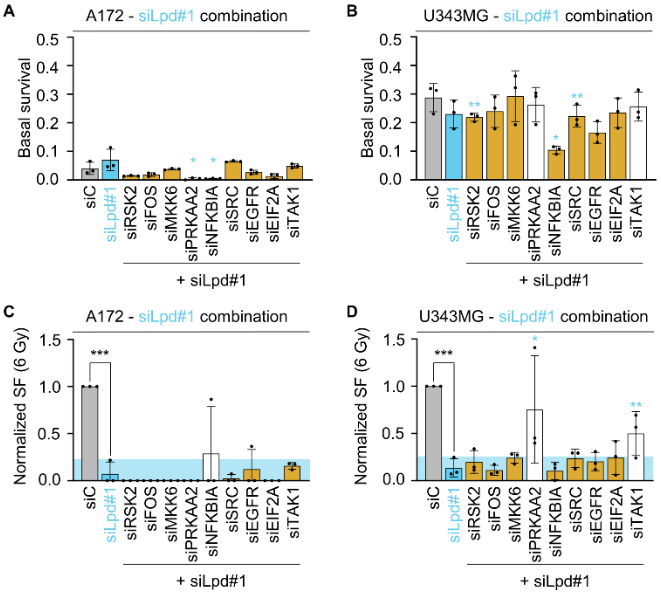

Figure S4. Lamellipodin and EGFR-MAPK signaling proteins co-regulate glioblastoma radiosensitivity. (**A**) Normalized clonogenic radiation survival (6 Gy) of A172 cells and (**B**) U343MG cells upon siRNA mediated knockdown of Lpd combined with the indicated proteins. (**C**) Normalized clonogenic radiation survival at 6 Gy of A172 and (**D**) U343MG cells upon siRNA-specific double knockdown of Lpd and indicated proteins. Yellow columns indicate a surviving fraction comparable to single Lpd knockdown, whereas white columns indicate a differing survival. The blue area shows the normalized surviving fraction upon Lpd silencing and 6 Gy X-ray exposure. Results present mean

± SD (n = 3, two-sided Student's t-test, siLpd / siC ***p < 0.001, siRNA / siLpd *p < 0.05, **p < 0.01). siRNA, small interfering RNA; C, control; Lpd, Lamellipodin; SF, surviving fraction.

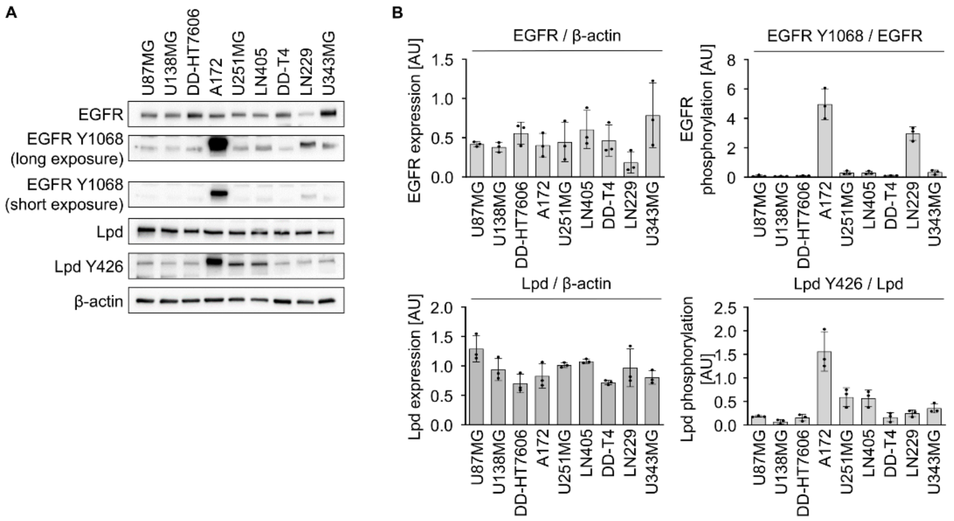

Figure S5 EGFR and Lpd expression and phosphorylation status in glioblastoma cells. (**A**) Representative Western blot of EGFR and Lpd expression and phosphorylation in whole cell lysates of indicated glioblastoma cell lines. β-actin served as loading control. (**B**) Densitometric analysis of EGFR and Lpd expression and EGFR Y1068 (short exposure) and Lpd Y426 phosphorylation from (A). Expression of EGFR and Lpd was normalized to β-actin. EGFR and Lpd phosphorylation were calculated by normalization to EGFR or Lpd expression. Results represent mean ± SD (n = 3).

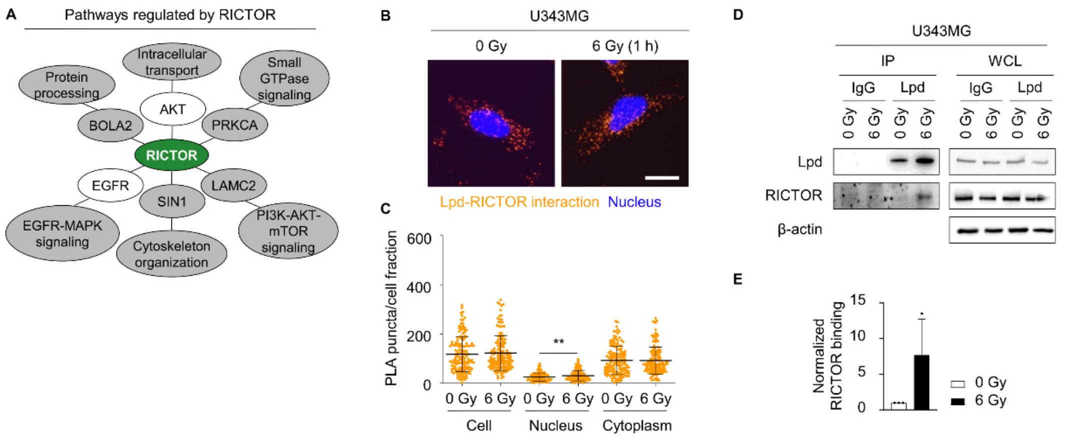

Figure S6. Lamellipodin and RICTOR interact. (**A**) Pathways regulated by RICTOR with connecting proteins identified by Lpd immunoprecipitation and mass spectrometry (grey) and published interaction partners of RICTOR (white). (**B**) Interaction of Lpd and RICTOR detected with proximity ligation assay in unirradiated and 6 Gy irradiated U343MG cells. Scale bar: 20 µm. (**C**) Quantification of puncta from (B) in whole cell, nucleus and cytoplasm using Fiji. Results present mean ± SD (n = 3, two-sided Student's t-test, n.s., not significant, *p < 0.05, **p < 0.01, ***p < 0.001). (**D**) Western blot showing expression of Lpd and RICTOR in immunoprecipitates and whole cell lysates 1 h after 0 Gy or 6 Gy irradiation from U343MG cells. (**E**) (**F**) Densitometric analyses presenting relative RICTOR binding from (E). Fold changes are calculated by normalization to Lpd and 0 Gy according to representative Western blot. Data are shown as mean ± SD (n =3). PLA, proximity ligation assay; IP, immunoprecipitation; WCL, whole cell lysates; IgG, immunoglobulin G; Lpd, Lamellipodin.

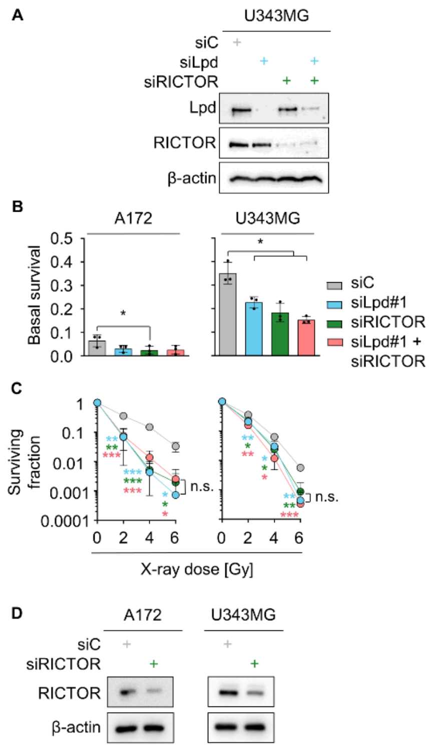

Figure S7. Lamellipodin and RICTOR co-control glioblastoma radiosensitivity and invasion. (**A**) Western blot of knockdown efficiency of control, Lpd and RICTOR specific siRNA in whole cell lysates of U343MG. (**B**) Basal survival (0 Gy) and (**C**) clonogenic radiation survival of A172 and U343MG upon Lpd- and RICTOR-specific siRNA knockdown. (**D**) Western Blot analysis of A172 and U343MG cells upon control and RICTOR knockdown. Results are mean ± SD (n = 3, two-sided Student's t-test, n.s., not significant, *p < 0.05, **p < 0.01, ***p < 0.001). siRNA, small interfering RNA; C, control; Lpd, Lamellipodin.

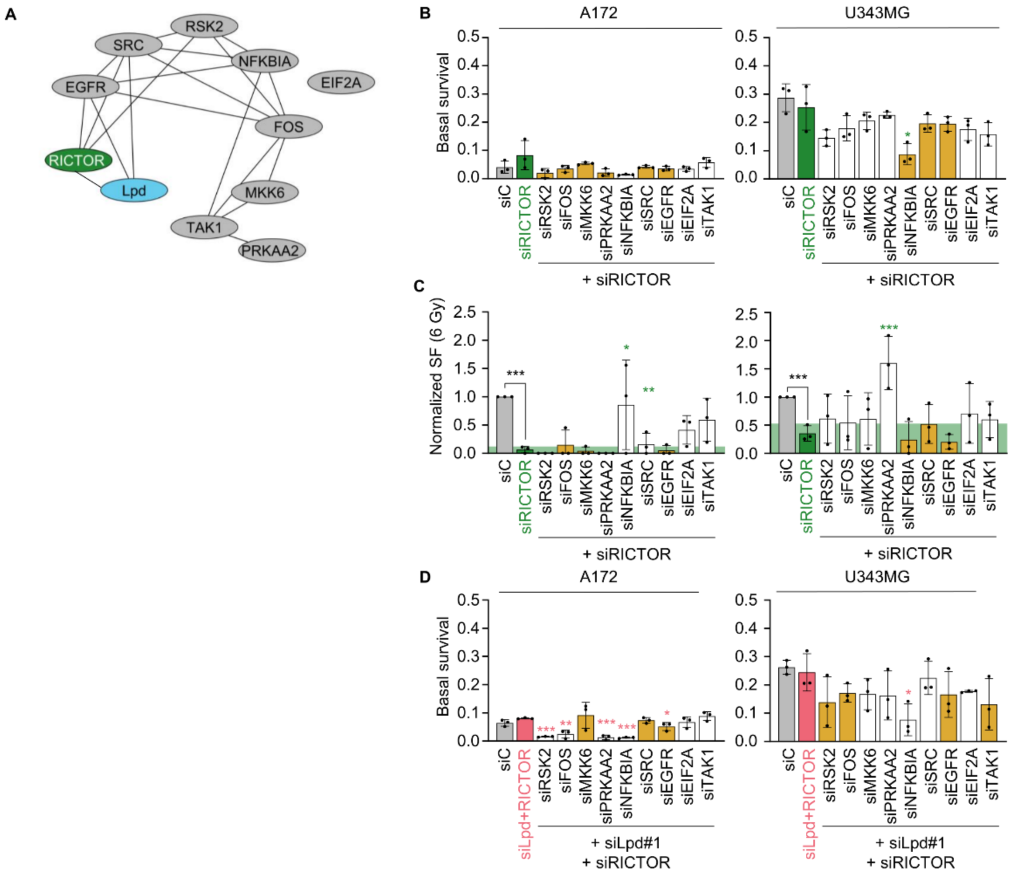

Figure S8. Lamellipodin and RICTOR jointly regulate radiosensitivity via EGFR. (**A**) Interactome of Lpd and RICTOR with the nine previous identified proteins EIF2A, EGFR, FOS, MKK6, NFKBIA, PRKAA2, RSK2, SRC and TAK1 using Cytoscape. (**B**) Basal survival (0 Gy) upon siRNA-specific double knockdown of RICTOR and the indicated proteins in A172 and U343MG cells. (**C**) Normalized surviving fraction at 6 Gy of A172 and U343MG among silencing of RICTOR and the indicated proteins. Basal survival (0 Gy) upon siRNA-specific triple knockdown of Lpd, RICTOR and indicated proteins in A172 and U343MG cells. (**D**) Basal survival (0 Gy) upon siRNA-specific triple knockdown of Lpd, RICTOR and indicated proteins in A172 and U343MG cells. Yellow columns indicate a surviving fraction comparable to single Lpd knockdown, whereas white columns indicate a differing survival compared to siRICTOR (B,C) and siLpd + siRICTOR (D). The green area in (C) represent the normalized surviving fraction upon siRICTOR and 6 Gy irradiation. Results show mean ± SD (n = 3, two-sided Student's t-test, siRICTOR / siC ***p < 0.001, siRNA / siRICTOR *p < 0.05, **p < 0.01, ***p < 0.001, siRNA / siLpd + siRICTOR *p < 0.05, **p < 0.01, ***p < 0.001). siRNA, small interfering RNA; C, control; Lpd, Lamellipodin; SF, surviving fraction

Supplementary Tables

Table S1. Synergistic and additive effects of siLpd knockdown and 6 Gy X-ray irradiation. Synergistic and additive effects were calculated as published [1] using following formula: S = r(a, b) – r(a0, b) x r(a, b0). a = siLpd; b = X-rays. Additive effects are shown in blue. Related to Figure 2.

Cell line	knockdown	r(a,b)	r(a0,b)	r(a,b0)	S
U87MG	siLpd#1	0.0753	0.0065	0.0493	0.0750
	siLpd#2	0.0954	0.0065	0.0392	0.0952
LN229	siLpd#1	0.0308	0.0190	0.1824	0.0273
	siLpd#2	0.0034	0.0190	0.1178	0.0012
DD-HT7606	siLpd#1	0.0904	0.0065	0.0328	0.0902
	siLpd#2	0.0784	0.0061	0.0294	0.0783
U138MG	siLpd#1	0.0170	0.0015	0.0071	0.0170
	siLpd#2	0.0795	0.0015	0.0182	0.0795
U251MG	siLpd#1	0.0485	0.0236	0.0834	0.0465
	siLpd#2	0.0972	0.0244	0.0883	0.0950
DD-T4	siLpd#1	0.0162	0.0162	0.0769	0.0150
	siLpd#2	0.0856	0.0213	0.1460	0.0825
LN405	siLpd#1	0.0290	0.0593	0.0658	0.0251
	siLpd#2	0.0023	0.0593	0.0144	0.0015
U343MG	siLpd#1	0.0052	0.0099	0.2383	0.0028
	siLpd#2	0.0049	0.0139	0.3550	0.0000
A172	siLpd#1	0.0008	0.0066	0.0567	0.0005
	siLpd#2	0.0017	0.0066	0.0205	0.0015

Table S2. Mean intensity of indicated phosphorylation sites at 0 Gy and 6 Gy upon siC and siLpd#1 treatment. Data are presented as mean ± SD. Related to Figure 3.

**Protein (Phosphosite)**	**siC 0 Gy**	**SD**	**siC 6 Gy**	**SD**	**siLpd#1 0 Gy**	**SD**	**siLpd#1 6 Gy**	**SD**
AKT (S473)	0.19	0.05	0.16	0.05	0.16	0.03	0.16	0.08
PRKAA2 (T172)	0.13	0.02	0.08	0.02	0.08	0.01	0.08	0.02
BAD (S112)	0.13	0.03	0.11	0.03	0.10	0.03	0.10	0.03
EIF4EBP1 (T36)	0.20	0.03	0.20	0.03	0.18	0.05	0.19	0.05
GSK3A (S21)	0.05	0.00	0.05	0.00	0.04	0.01	0.04	0.01
GSK3B (S9)	0.34	0.07	0.31	0.07	0.27	0.05	0.25	0.08
mTOR (S2448)	0.14	0.04	0.12	0.04	0.12	0.04	0.13	0.05
CDKN1B (T198)	0.05	0.03	0.07	0.03	0.05	0.02	0.05	0.02
P70S6K (T421/S424)	0.16	0.02	0.15	0.02	0.15	0.03	0.16	0.04
PDK1 (S241)	0.12	0.01	0.11	0.01	0.12	0.03	0.12	0.04
PRAS40 (T246)	0.08	0.02	0.09	0.02	0.06	0.02	0.07	0.03
PTEN (S380)	0.13	0.05	0.11	0.05	0.10	0.01	0.11	0.02
RAF1 (S301)	0.07	0.03	0.06	0.03	0.05	0.01	0.05	0.03
RPS6 (S235/236)	0.19	0.08	0.16	0.08	0.15	0.06	0.16	0.07
EGFR (Ser1070)	0.16	0.07	0.15	0.07	0.19	0.12	0.13	0.09
JAK1 (Tyr1022)	0.07	0.02	0.05	0.02	0.06	0.01	0.06	0.01
JAK2 (Tyr1007/1008)	0.07	0.02	0.06	0.02	0.07	0.01	0.06	0.02
SHP1 (Ser591)	0.02	0.00	0.02	0.00	0.02	0.00	0.01	0.01
SHP2 (Tyr542)	0.12	0.03	0.12	0.03	0.11	0.01	0.09	0.01
Src (Tyr419)	0.06	0.01	0.07	0.01	0.06	0.01	0.05	0.01
Stat1 (Ser727)	0.02	0.01	0.02	0.01	0.03	0.00	0.03	0.00
Stat2 (Tyr689)	0.06	0.01	0.06	0.01	0.06	0.01	0.06	0.00
Stat3 (Tyr705)	0.04	0.01	0.04	0.01	0.05	0.01	0.04	0.00
Stat5 (Tyr694)	0.01	0.00	0.02	0.00	0.02	0.00	0.02	0.00
Stat6 (Tyr641)	0.01	0.00	0.01	0.00	0.01	0.00	0.01	0.00
TYK2 (Tyr1054)	0.06	0.01	0.07	0.01	0.05	0.01	0.06	0.01
CREB (S133)	0.13	0.03	0.12	0.03	0.08	0.03	0.05	0.01
ERK1/2 (T202/Y204) (Y185/Y187)	0.07	0.01	0.08	0.01	0.07	0.01	0.06	0.02
HSP27 (S82)	0.06	0.02	0.06	0.02	0.05	0.02	0.04	0.01
JNK (T183)	0.18	0.05	0.15	0.05	0.11	0.07	0.09	0.04
MEK (S217/221)	0.04	0.02	0.03	0.02	0.03	0.01	0.02	0.00
MKK3 (S189)	0.12	0.02	0.11	0.02	0.08	0.05	0.06	0.02
MMK6 (S207)	0.04	0.01	0.04	0.01	0.02	0.02	0.02	0.01
MSK2 (S360)	0.03	0.01	0.03	0.01	0.03	0.00	0.02	0.00

MAPK14 (T180/Y182)	0.10	0.02	0.10	0.02	0.10	0.02	0.10	0.01
TP53 (S15)	0.23	0.05	0.19	0.05	0.16	0.10	0.14	0.05
RSK1 (S380)	0.05	0.02	0.06	0.02	0.03	0.02	0.03	0.01
RSK2 (S386)	0.04	0.02	0.04	0.02	0.02	0.01	0.02	0.01
ATM (S1981)	0.25	0.01	0.25	0.01	0.25	0.06	0.21	0.05
EIF2A (S51)	0.04	0.01	0.04	0.01	0.04	0.01	0.03	0.01
HDAC2 (S394)	0.18	0.01	0.19	0.01	0.18	0.05	0.17	0.03
HDAC4 (S632)	0.11	0.02	0.13	0.02	0.10	0.01	0.11	0.04
NFKB1 (S32)	0.04	0.01	0.03	0.01	0.03	0.01	0.04	0.01
MSK1 (S376)	0.05	0.01	0.05	0.01	0.04	0.01	0.05	0.01
NFKBIA (S536)	0.09	0.01	0.10	0.01	0.09	0.01	0.09	0.01
TAK1 (S412)	0.02	0.01	0.04	0.01	0.03	0.02	0.02	0.01
TBK1 (S172)	0.83	0.04	0.89	0.04	0.88	0.03	0.81	0.10
ZAP70 (Y292)	0.06	0.01	0.09	0.01	0.07	0.02	0.08	0.01
ATF2 (T69/71)	0.05	0.02	0.05	0.02	0.04	0.01	0.05	0.01
FOS (T232)	0.02	0.01	0.02	0.01	0.01	0.00	0.02	0.00
JUN (S73)	0.09	0.01	0.07	0.01	0.08	0.03	0.07	0.01
SMAD1 (S463/465)	0.03	0.01	0.03	0.01	0.02	0.00	0.02	0.00
SMAD2 (S245/250/255)	0.03	0.02	0.03	0.02	0.04	0.01	0.03	0.02
SMAD4 (T277)	0.07	0.02	0.06	0.02	0.06	0.01	0.06	0.01
SMAD5 (S463/465)	0.05	0.02	0.05	0.02	0.05	0.01	0.05	0.00

Table S3. Fold change normalized to 0 Gy of indicated phosphorylation sites calculated from mean intensity from Table S1. Data are presented as mean ± SD. Related to Figure 3.

Protein (Phosphosite)	siC 6 Gy / 0 Gy	SD	siLpd#1 6 Gy / 0 Gy	SD
AKT (S473)	0.90	0.50	0.93	0.38
PRKAA (T172)	0.60	0.11	1.00	0.39
BAD (S112)	0.86	0.12	0.98	0.10
EIF4EBP1 (T36)	0.98	0.11	1.02	0.09
GSK3A (S21)	1.09	0.44	1.17	0.16
GSK3B (S9)	0.89	0.23	0.91	0.17
mTOR (S2448)	0.87	0.10	1.04	0.16
CDKN1B (T198)	2.64	3.47	0.93	0.24
P70S6K (T421/S424)	0.94	0.14	1.10	0.19
PDK1 (S241)	0.93	0.26	1.05	0.11
PRAS40 (T246)	1.30	0.41	1.25	0.23
PTEN (S380)	0.93	0.13	1.08	0.18
RAF1 (S301)	0.88	0.40	0.90	0.29
RPS6 (S235/236)	0.84	0.12	1.02	0.12
EGFR (Ser1070)	0.98	0.19	0.64	0.12
JAK1 (Tyr1022)	0.87	0.28	1.04	0.38
JAK2 (Tyr1007/1008)	0.81	0.08	0.90	0.33
SHP1 (Ser591)	0.85	0.16	0.84	0.32
SHP2 (Tyr542)	1.09	0.59	0.85	0.09
Src (Tyr419)	1.39	0.23	0.93	0.11
Stat1 (Ser727)	1.18	0.90	0.98	0.08
Stat2 (Tyr689)	1.04	0.31	1.05	0.15
Stat3 (Tyr705)	1.00	0.14	0.98	0.16
Stat5 (Tyr694)	1.15	0.51	1.13	0.17
Stat6 (Tyr641)	1.61	1.08	1.13	0.27
TYK2 (Tyr1054)	1.17	0.42	1.39	0.51
CREB (S133)	0.99	0.23	0.76	0.15
ERK1/2 (T202/Y204) (Y185/Y187)	1.05	0.21	0.80	0.20
HSP27 (S82)	1.07	0.25	0.99	0.38
JNK (T183)	0.85	0.11	0.94	0.43
MEK (S217/221)	1.04	0.34	1.07	0.35
MKK3 (S189)	0.99	0.15	0.96	0.28
MMK6 (S207)	0.94	0.13	2.11	1.98
MSK2 (S360)	0.98	0.44	0.91	0.37
MAPK14 (T180/Y182)	1.01	0.11	1.01	0.08
TP53 (S15)	0.84	0.21	1.06	0.43
RSK1 (S380)	1.10	0.18	1.04	0.39
RSK2 (S386)	0.83	0.38	1.87	1.45
ATM (S1981)	1.01	0.13	0.86	0.24
EIF2A (S51)	1.00	0.08	0.65	0.13
HDAC2 (S394)	1.05	0.14	1.07	0.55
HDAC4 (S632)	1.14	0.11	1.06	0.36
NFKB1 (S32)	0.78	0.22	1.21	0.22
MSK1 (S376)	0.99	0.22	1.04	0.23
NFKBIA (S536)	1.12	0.07	1.00	0.14
TAK1 (S412)	3.44	3.61	0.89	0.20

TBK1 (S172)	1.07	0.07	0.92	0.10
ZAP70 (Y292)	1.65	0.52	1.38	0.76
ATF2 (T69/71)	1.02	0.14	1.22	0.27
FOS (T232)	0.96	0.28	2.05	0.55
JUN (S73)	0.81	0.13	0.95	0.31
SMAD1 (S463/465)	1.07	0.13	1.03	0.24
SMAD2 (S245/250/255)	1.60	0.92	0.71	0.29
SMAD4 (T277)	1.10	0.46	0.97	0.25
SMAD5 (S463/465)	1.17	0.45	1.02	0.21

Table S4. Lamellipodin bound proteins detected by mass spectrometry following Lamellipodin immunoprecipitation from unirradiated cells (IgG as non-specific control). Related to Figure 5.

Protein	Ratio of mean Log2 LFQ intensity (Lpd / IgG)	Ratio of mean peptides (IgG / Lpd)
ADD3	12.6	2.0
ADGRG1;GPR56	12.1	3.0
ARPC1A	13.0	2.0
BCL7C	13.7	2.0
BOLA2;BOLA2B	12.4	3.0
CEP85	25.9	4.3
CLPTM1L	12.6	2.0
COG3	12.4	2.0
COL1A1	12.6	2.0
COX4I1	12.7	3.0
DENR	23.2	4.0
DFFA	12.5	3.0
DMBT1	12.6	2.0
ESYT2	12.0	2.0
GTF3C3	11.5	2.0
HS1BP3	12.3	3.0
LAMC2	12.7	2.0
MAPRE3	12.0	2.0
MPZL1	13.2	2.0
MTR	11.4	2.0
NUCB1	23.6	2.0
OBSL1	12.2	3.0
PRAF2	12.6	2.0
PRIM1	10.8	2.0
PTMA	12.4	3.0
R3HDM1	23.6	2.0
RAF1;ARAF	12.7	2.0
RICTOR	2.0	3.5
S100A13	24.3	4.0
SEC61A1	13.1	2.0
SIPA1L3	12.5	2.5
SNX7	11.7	2.0
SP1	12.8	2.0
TRMT1L	12.5	2.0
ZFR	11.7	2.0
ZMPSTE24	12.8	3.0

Table S5. Lamellipodin bound proteins detected by mass spectrometry following Lamellipodin immunoprecipitation from 6-Gy irradiated cells (IgG as non-specific control). Related to Figure 5.

Protein	Ratio of mean Log2 LFQ intensity (Lpd / IgG)	Ratio of mean peptides (IgG / Lpd)
ABHD14B	23.9	4.0
ABLIM3	11.8	2.0
ABR;BCR	12.0	2.0
ADAR	24.0	2.5
AGO4	12.6	2.0
AKAP2	12.3	2.0
ANAPC1	11.0	2.0
ANAPC4	11.5	2.0
ANKS1A	11.0	2.0
ANXA6	12.9	3.0
APOBEC3G;APOBEC3F	11.5	2.0
ARG1	12.7	2.0
ARL8B;ARL8A	23.6	2.0
ARPC1A	13.6	2.0
ATG16L1	11.9	2.0
BRE	11.9	2.0
CCDC50	24.8	2.5
CFAP36	11.6	2.0
CKB	12.1	2.0
CLTCL1	12.4	2.0
CNIH4	12.3	2.0
CNOT3	24.1	2.0
CNOT7	12.0	2.0
CNTNAP1	12.5	5.0
COL1A1	13.1	2.0
COMMD3;COMMD3-BMI1	12.0	2.0
CWF19L1	11.6	2.0
CYFIP2	11.9	2.0
DCD	10.9	2.0
DENND1A	2.0	3.8
DHPS	24.4	2.0
DHX32	10.8	2.0
DHX57	13.0	3.5
DICER1	10.7	2.0
DYNC1I1	13.2	2.0
DYRK1A;DYRK1B	12.4	2.0
EDC3	24.2	2.3
ELMO2;ELMO1	23.3	2.0
EPS8	12.1	2.0
FADS3	11.6	2.0
FAM65A	12.8	4.0
FAM91A1	12.0	2.0
FANCI	11.8	3.0
FKBP8	25.6	3.5
FRMD4A	12.4	4.0
FRYL	11.6	2.0

G6PC3	12.2	2.0
GALE	12.5	2.0
GALNT7	11.2	2.0
GEMIN4	12.3	3.0
GMFB	11.5	2.0
GNE	23.5	2.5
GPSM2	12.3	2.0
GTF2F2	12.2	2.0
GTF3C1	12.0	2.0
GTF3C3	11.7	3.0
GTF3C4	12.9	2.0
GTPBP1	12.1	2.0
GYS1	12.1	2.0
HEATR3	11.3	2.0
HELZ	11.2	2.0
HELZ2	24.1	2.0
HNRNPK	12.3	2.0
HSPE1-MOB4	12.3	2.0
INPP5K	12.0	2.0
ITPA	23.6	2.0
ITSN2	11.8	3.0
KIAA0196	12.5	5.0
KIDINS220	11.7	2.0
KIF2C	23.8	2.0
KLHDC10	11.6	2.0
LIMD1	11.8	3.0
M6PR	12.3	2.0
MALT1	22.9	2.0
MAPK10;MAPK9	22.3	2.0
MAPKAP1	25.4	2.0
MASTL	11.5	2.0
MED14	12.5	2.5
MED16	12.1	2.0
METTL1	11.2	2.0
MMTAG2	11.8	3.0
MORC3	11.6	2.0
MPRIP	13.0	2.0
MPST	12.2	2.0
MRPL37	24.5	2.0
MRPL40	11.3	2.0
MRPL47	12.3	3.0
MTMR9	11.8	3.0
MVD	12.3	3.0
MYLK	25.9	2.0
NAF1	12.0	2.0
NELFCD;TH1L	12.3	2.0
NFATC2IP	11.7	2.0
NHLRC2	24.1	2.0
NMD3	12.1	3.0
NUF2	24.5	3.0
NUP205	11.9	2.0
NUP43	11.5	2.0

NUP85	12.5	2.0
OSBPL11;OSBPL10	12.5	3.0
PAK4	12.3	3.0
PCDHGC3	12.2	2.0
PI4KA	12.9	2.0
PLEC	12.4	2.0
POFUT2	12.0	2.0
POP1	12.2	2.0
PREPL	12.3	2.0
PRKCA;PRKCB	12.6	3.0
PRKRA	12.7	2.0
PRPS1	12.6	2.0
PSMF1	11.5	2.0
PSMG2	13.1	4.0
PSPH	11.6	2.0
PTPN9	11.8	2.0
R3HDM1	12.3	3.0
RAB35	12.3	3.0
RAP2B	13.0	2.0
RAPH1	28.1	2.0
RBM42	12.2	2.0
RICTOR	2.1	3.1
SCD5	11.2	2.0
SLC25A10	12.4	2.0
SLC35F6	10.5	2.0
SLC38A2	12.7	2.0
SLC7A1	11.8	2.0
SMCHD1	11.9	2.0
SMG9	12.7	3.0
SPATA5	23.9	2.5
SSH1	24.0	2.0
STARD13	12.4	2.0
STUB1	12.5	2.0
SYDE1	23.9	2.0
SYNJ1	23.8	3.3
TACO1	12.1	2.0
TAP2	12.0	2.0
TEX10	11.1	2.0
TMEM205	12.4	2.0
TRMT10C	12.1	2.0
TSSC1	11.7	2.0
TTC7B	11.8	2.0
UBAP2	12.2	2.0
USP34	10.8	2.0
UXS1	11.8	2.0
VAC14	24.4	2.0
VPS41	12.6	2.0
WWP2	11.2	2.0
XAB2	12.0	2.0
XRN1	11.2	2.0
ZNF316	11.2	2.0

References

1. Yan, H.; Zhang, B.; Li, S.; Zhao, Q. A formal model for analyzing drug combination effects and its application in TNF-alpha-induced NFkappaB pathway. *BMC Syst Biol* **2010**, *4*, 50, doi:10.1186/1752-0509-4-50.
