# Supplementary figures and images for: Lamellipodin-RICTOR Signaling Mediates Glioblastoma Cell Invasion and Radiosensitivity Downstream of EGFR
Source: Cancers (Basel). 2021 Oct 24;13(21):5337. doi: 10.3390/cancers13215337 (PMC8582497; doi:10.3390/cancers13215337)

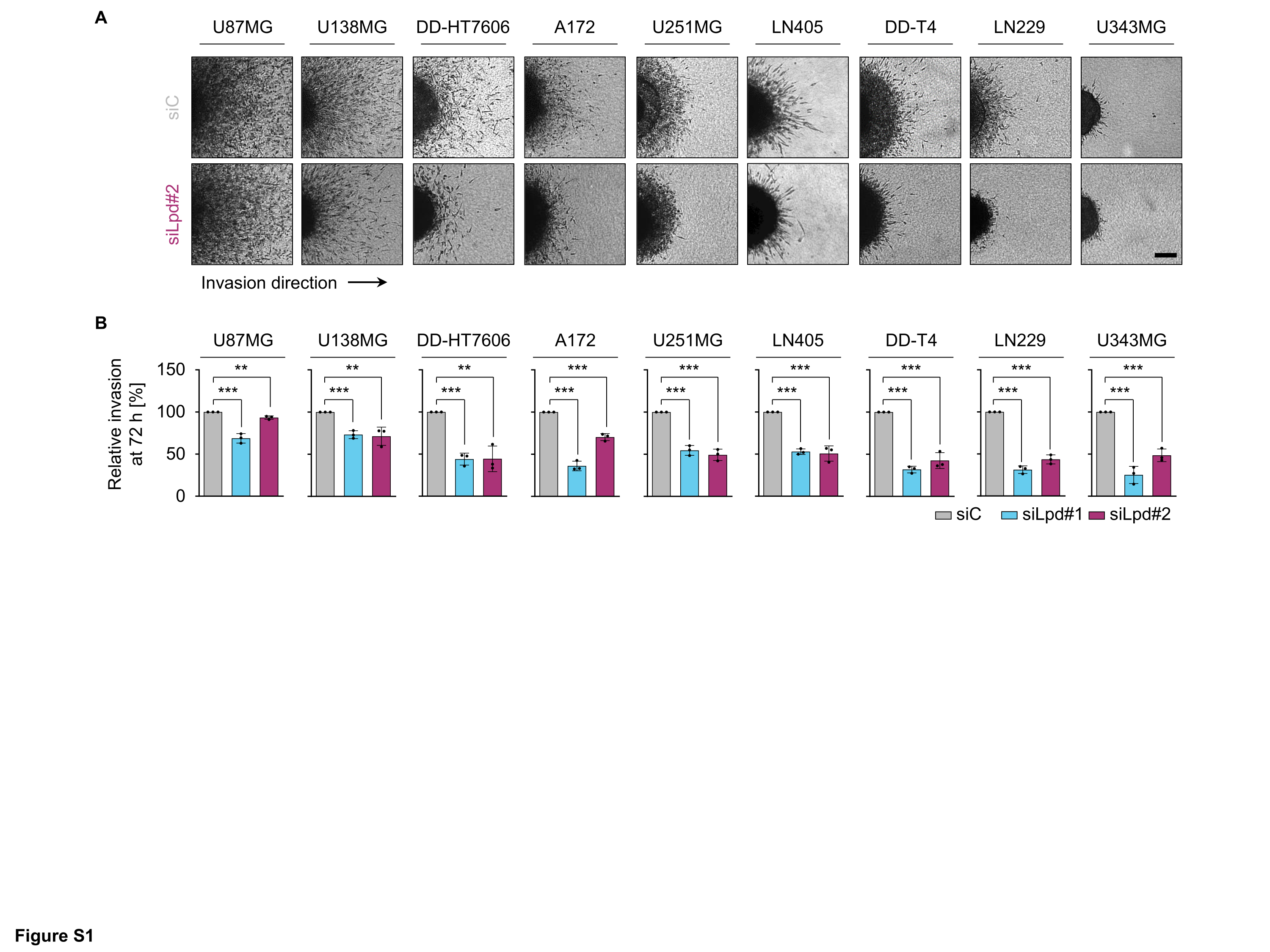

Supplement: Supplementary file 1 [file cancers-13-05337-s001.zip › cancers-1386069 supplementary/Moritz et al_Figure S1.tif]

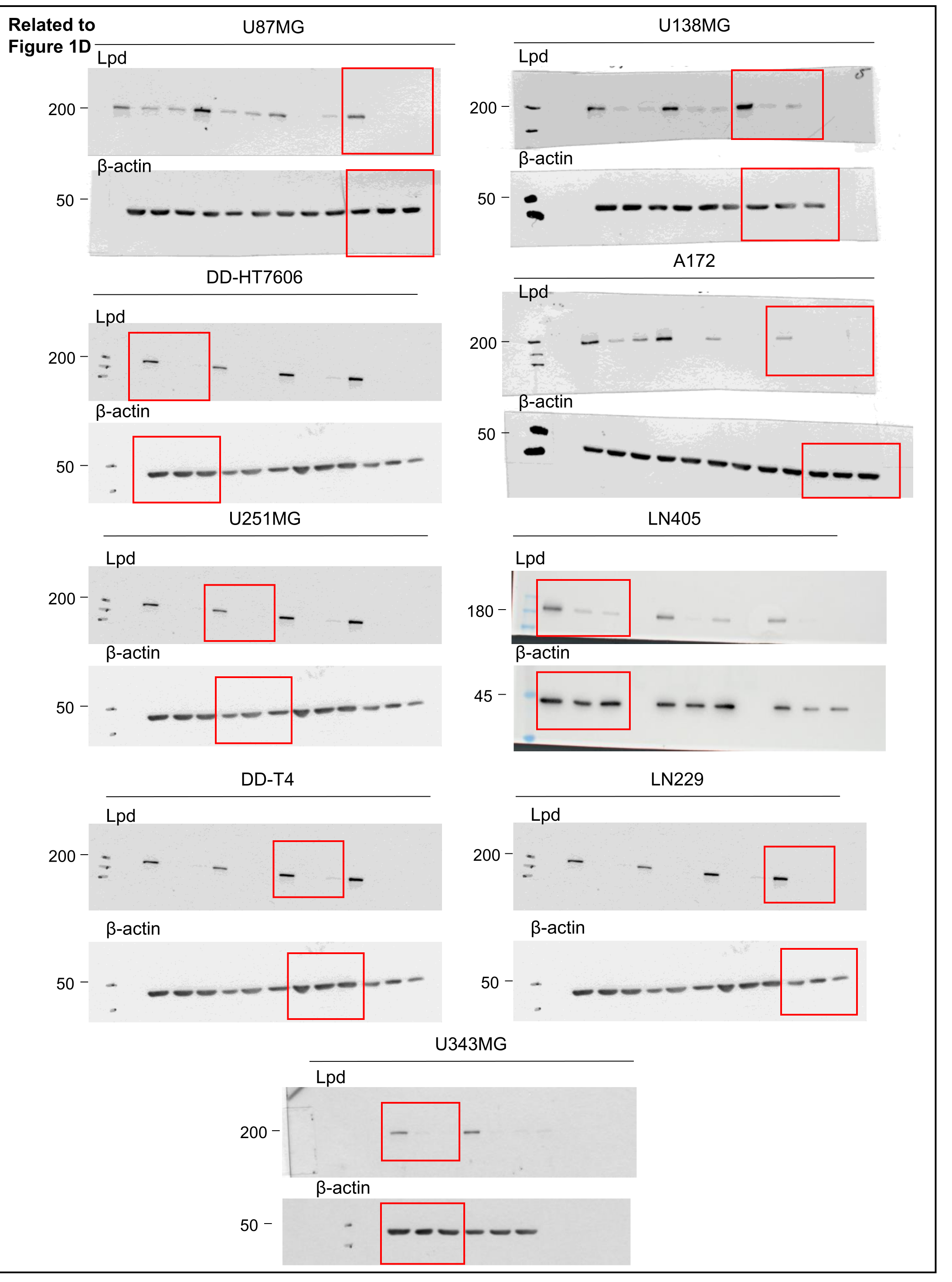

Supplement: Supplementary file 1 [file cancers-13-05337-s001.zip › cancers-1386069 supplementary/Moritz et al_Figure S10.tif]

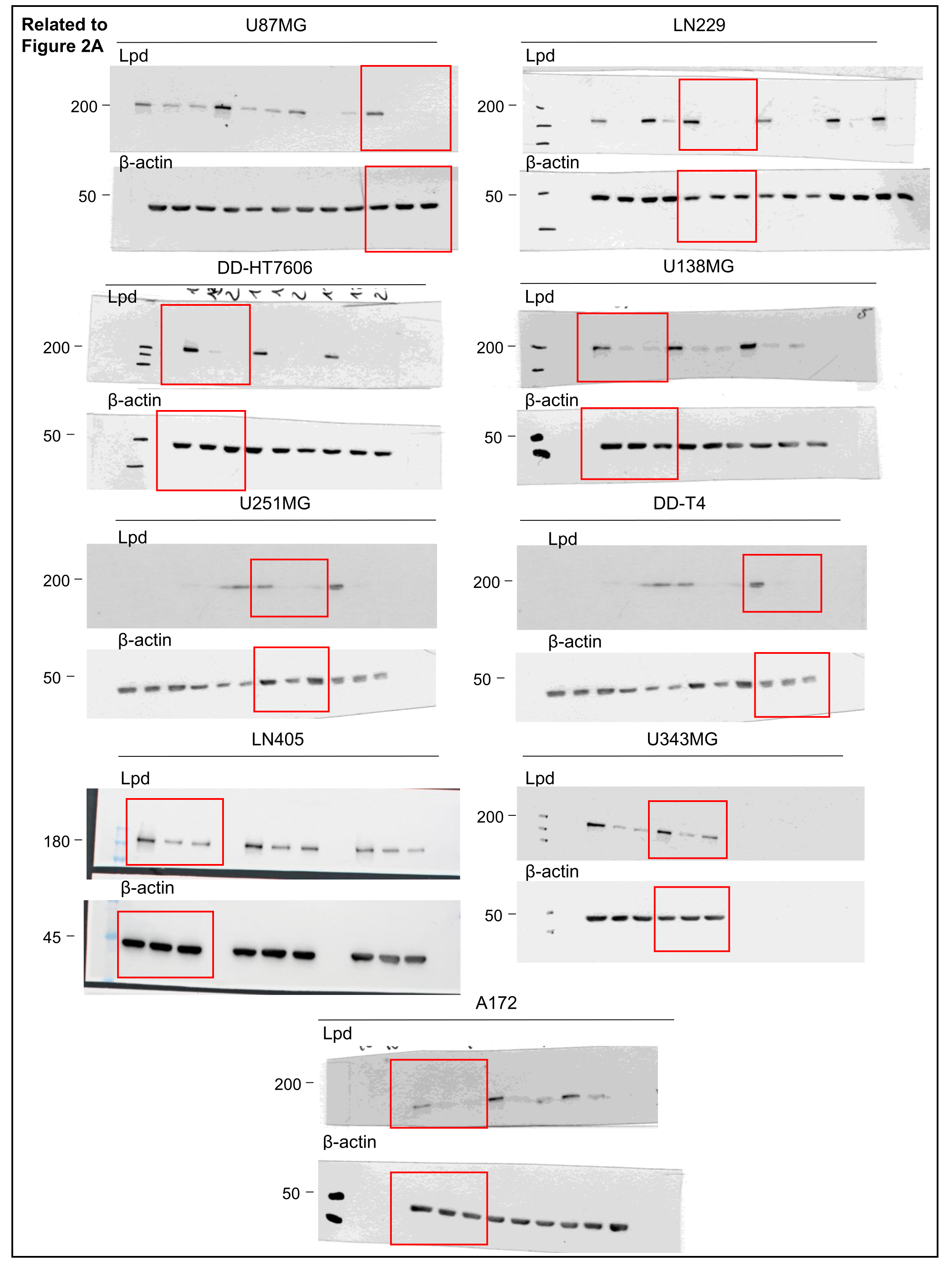

Supplement: Supplementary file 1 [file cancers-13-05337-s001.zip › cancers-1386069 supplementary/Moritz et al_Figure S11.tif]

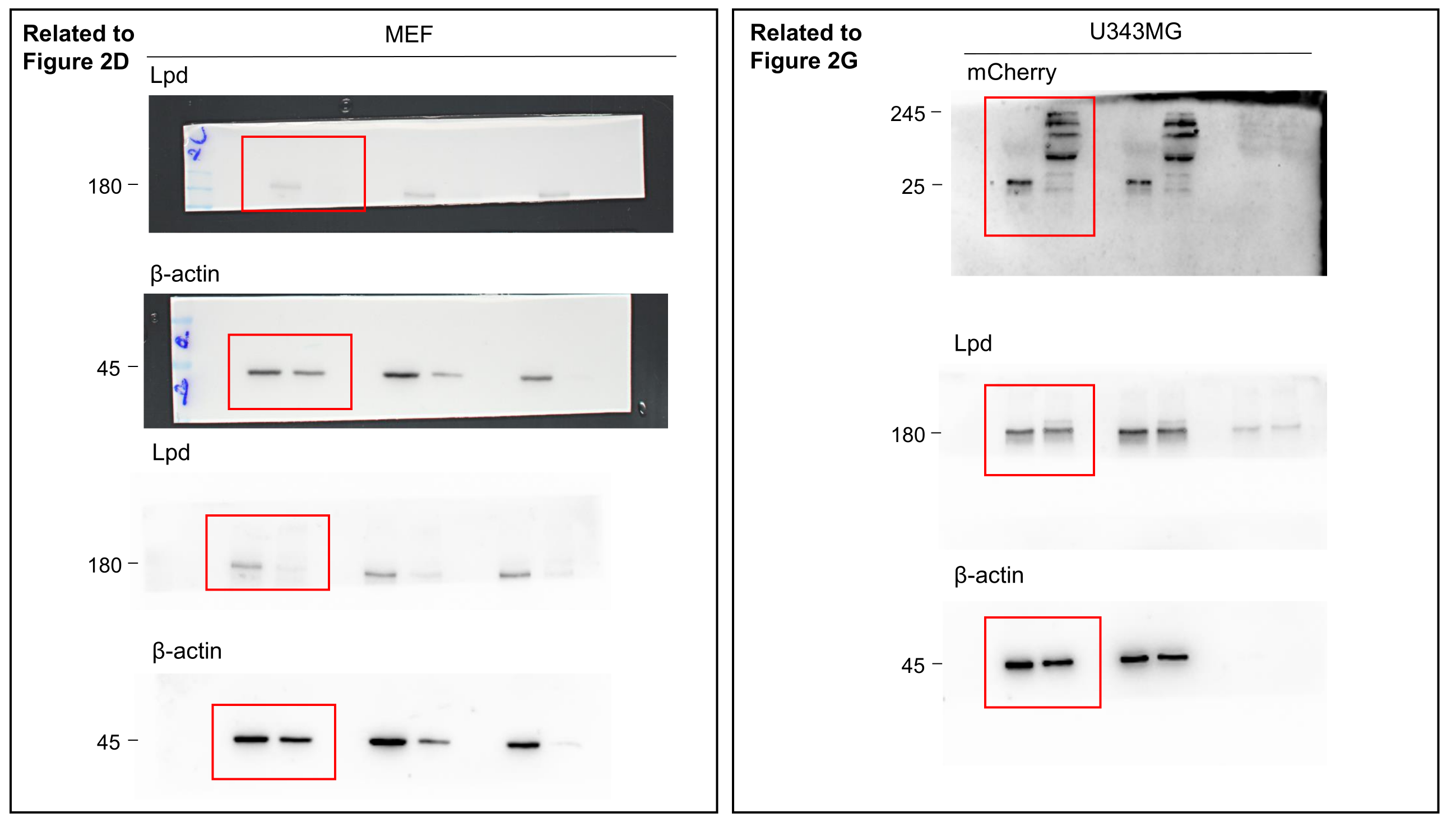

Supplement: Supplementary file 1 [file cancers-13-05337-s001.zip › cancers-1386069 supplementary/Moritz et al_Figure S12.tif]

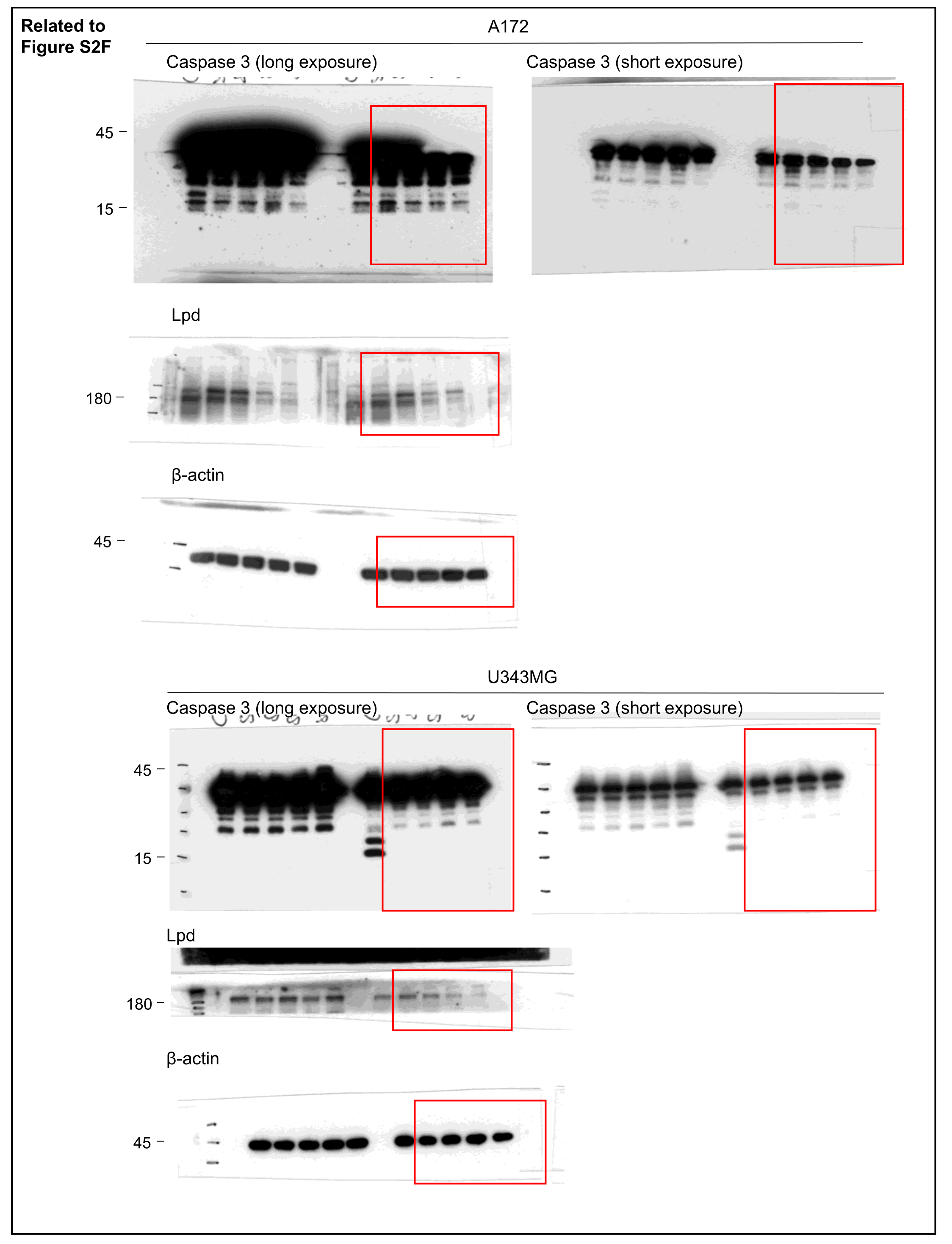

Supplement: Supplementary file 1 [file cancers-13-05337-s001.zip › cancers-1386069 supplementary/Moritz et al_Figure S13.tif]

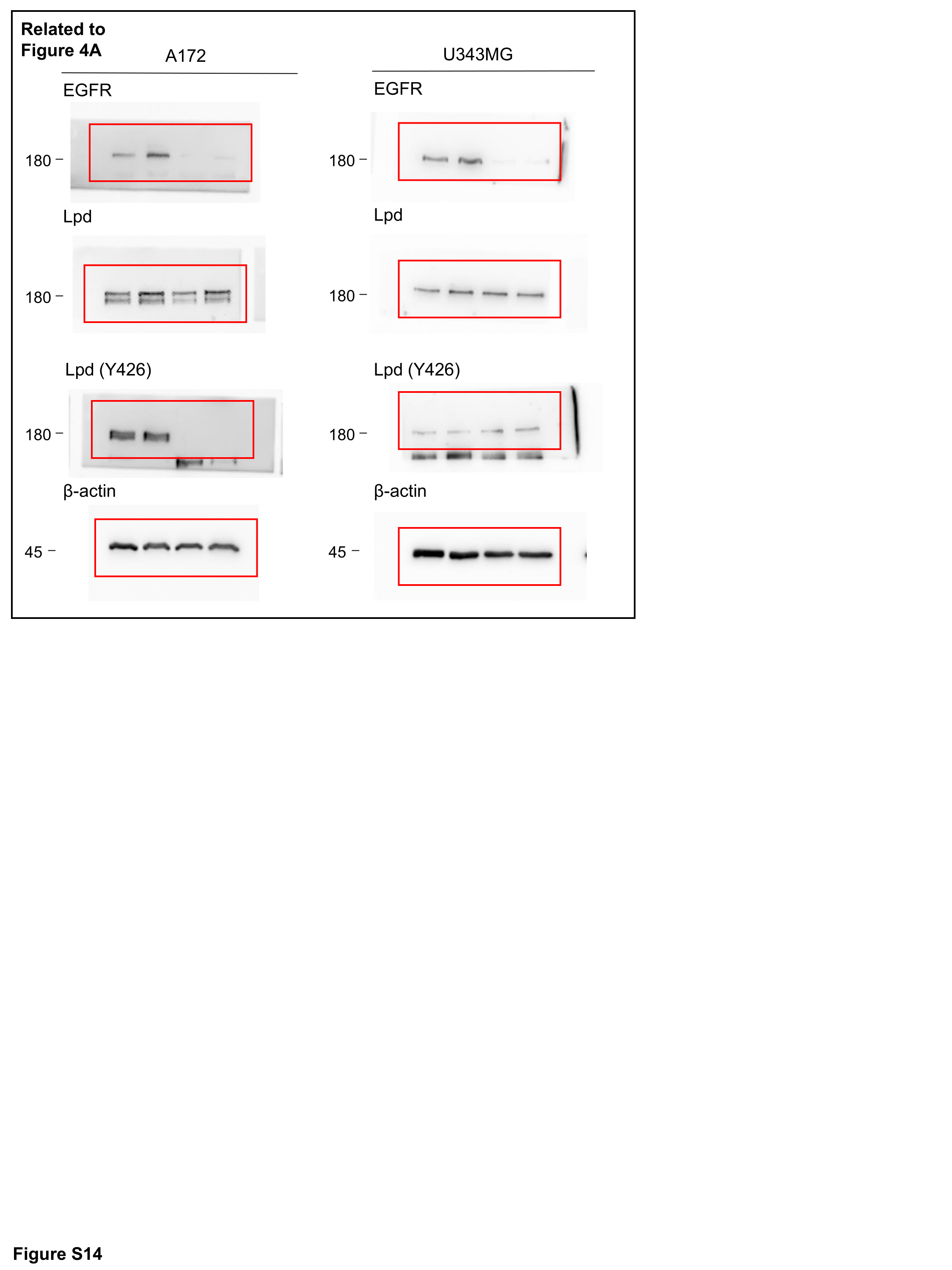

Supplement: Supplementary file 1 [file cancers-13-05337-s001.zip › cancers-1386069 supplementary/Moritz et al_Figure S14.tif]

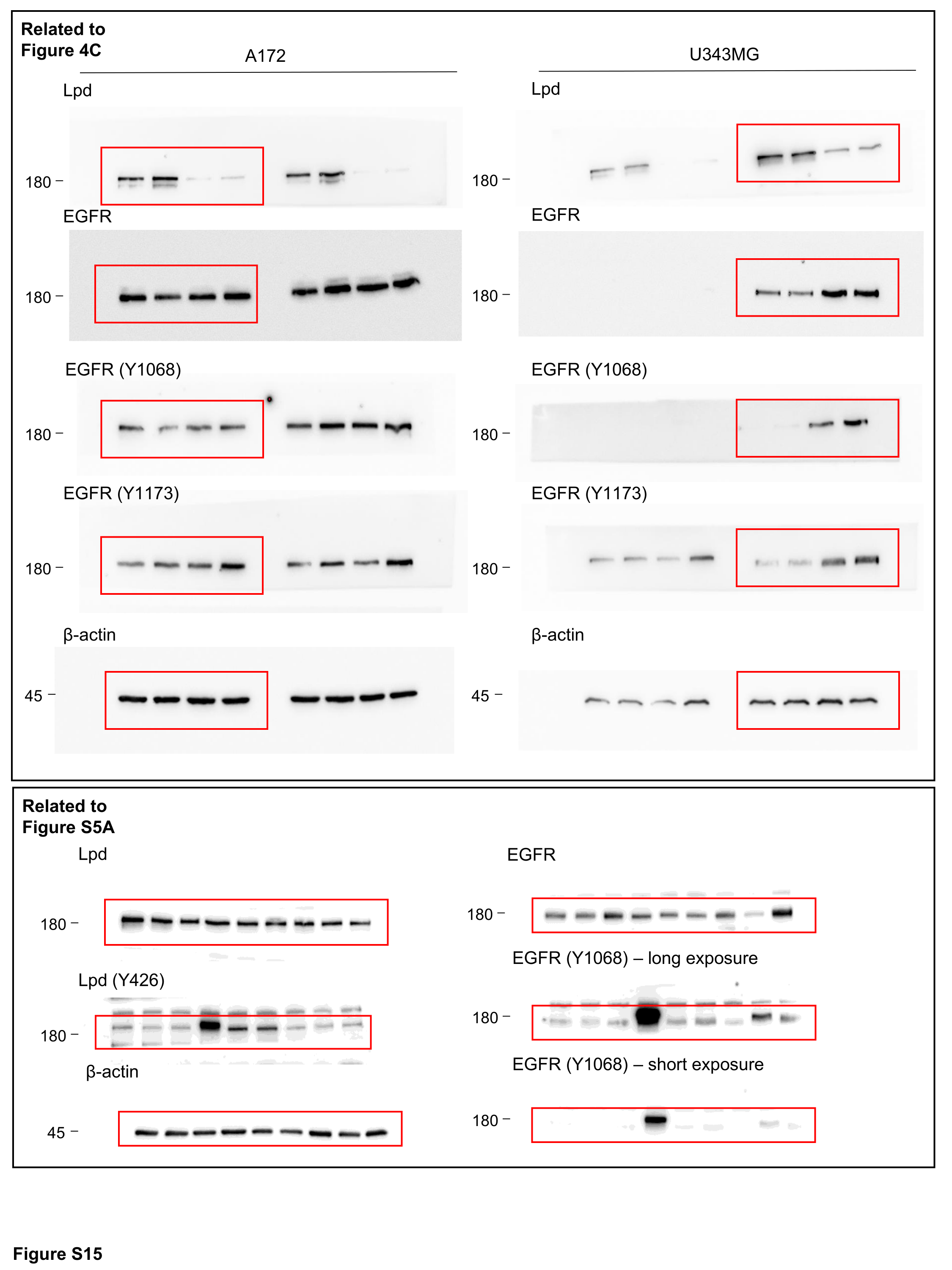

Supplement: Supplementary file 1 [file cancers-13-05337-s001.zip › cancers-1386069 supplementary/Moritz et al_Figure S15.tif]

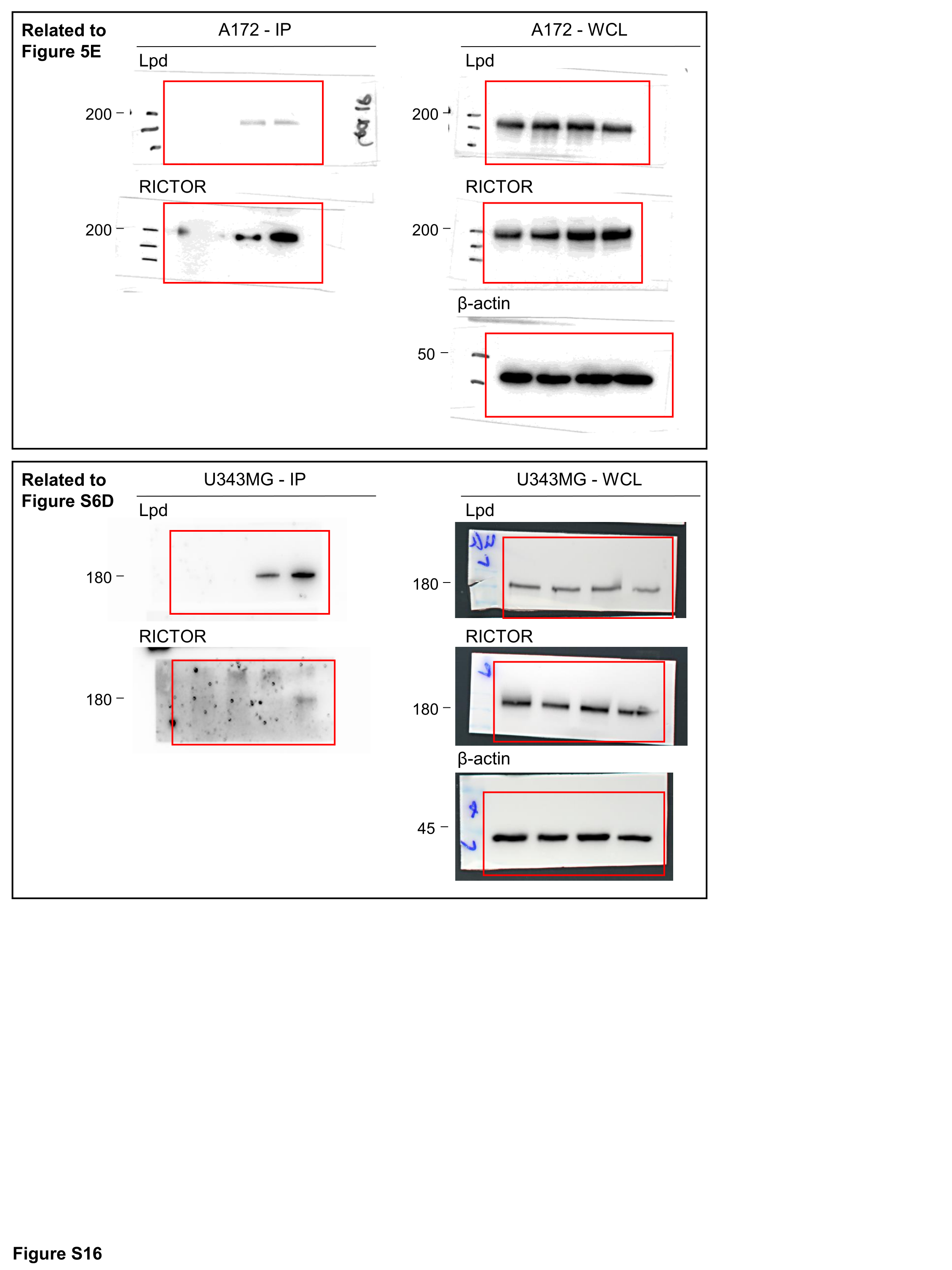

Supplement: Supplementary file 1 [file cancers-13-05337-s001.zip › cancers-1386069 supplementary/Moritz et al_Figure S16.tif]

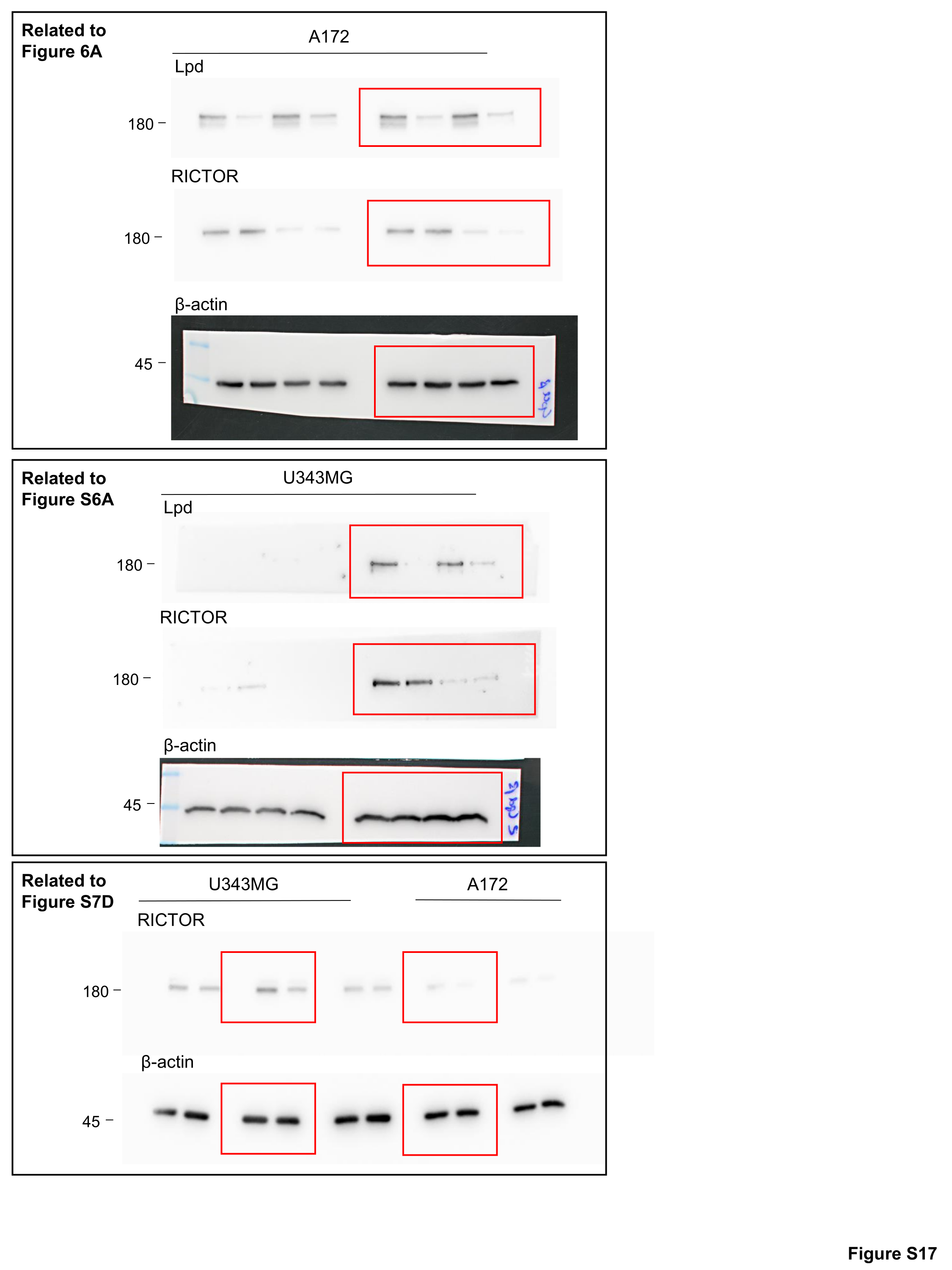

Supplement: Supplementary file 1 [file cancers-13-05337-s001.zip › cancers-1386069 supplementary/Moritz et al_Figure S17.tif]

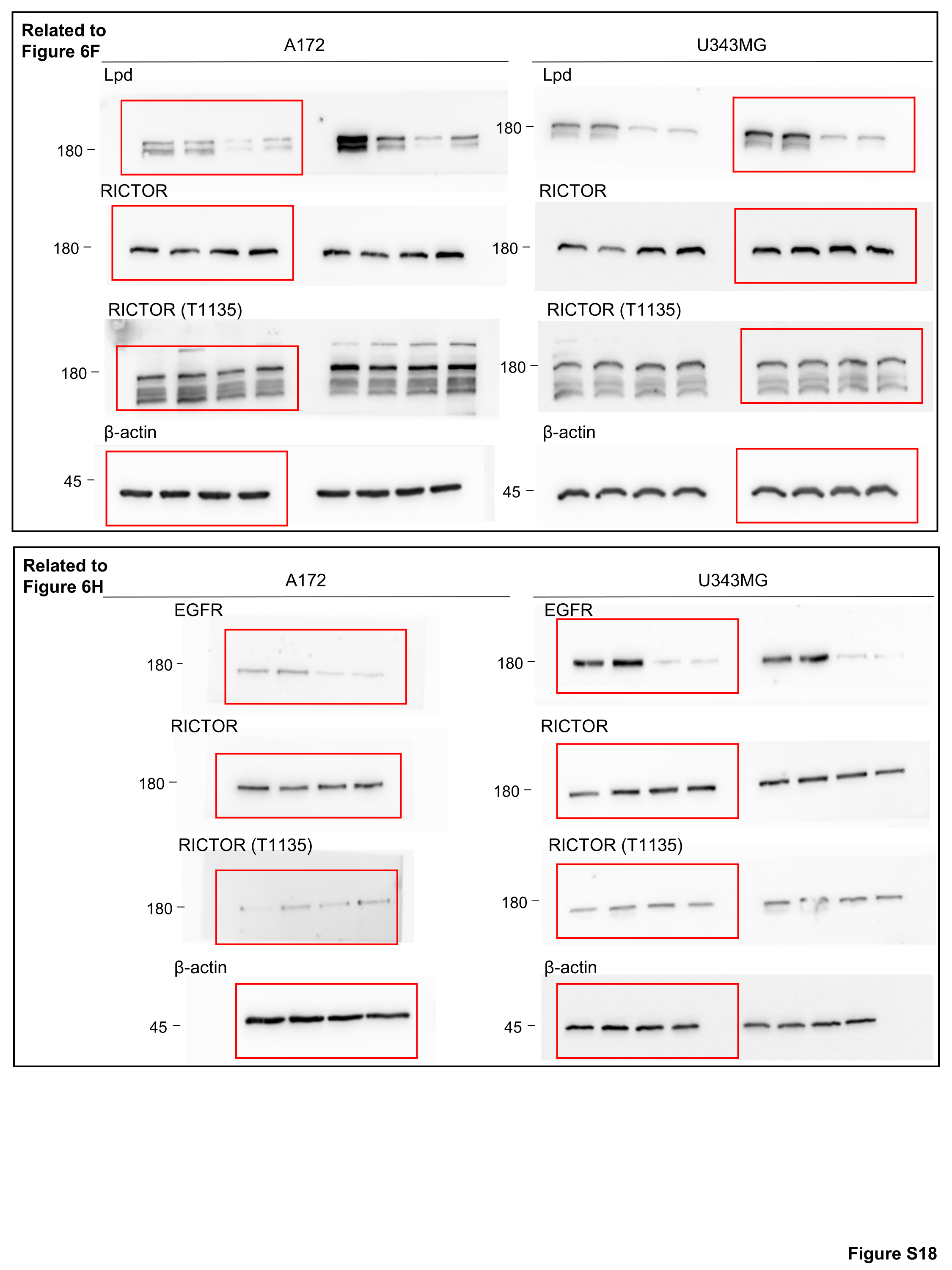

Supplement: Supplementary file 1 [file cancers-13-05337-s001.zip › cancers-1386069 supplementary/Moritz et al_Figure S18.tif]

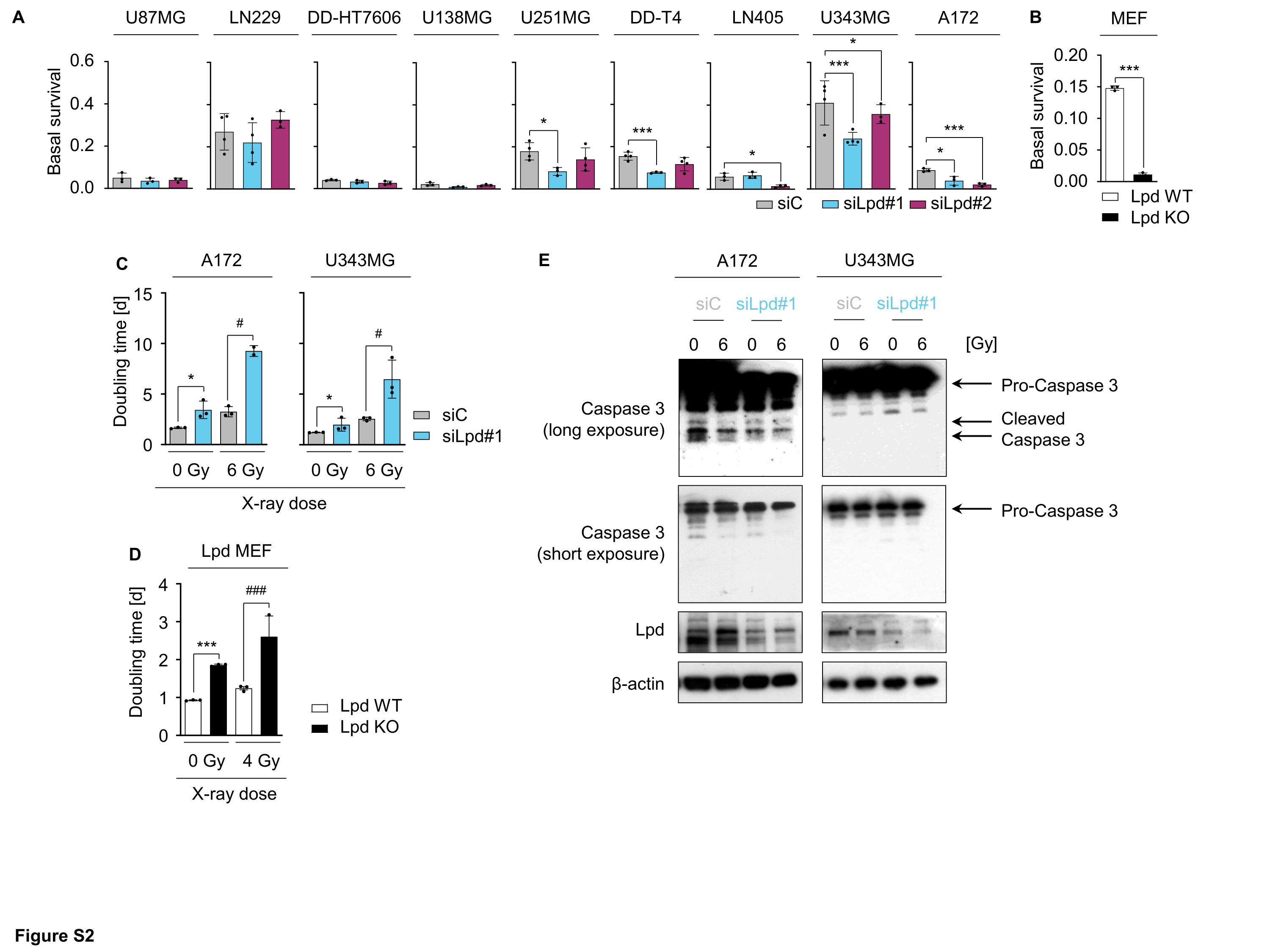

Supplement: Supplementary file 1 [file cancers-13-05337-s001.zip › cancers-1386069 supplementary/Moritz et al_Figure S2.tif]

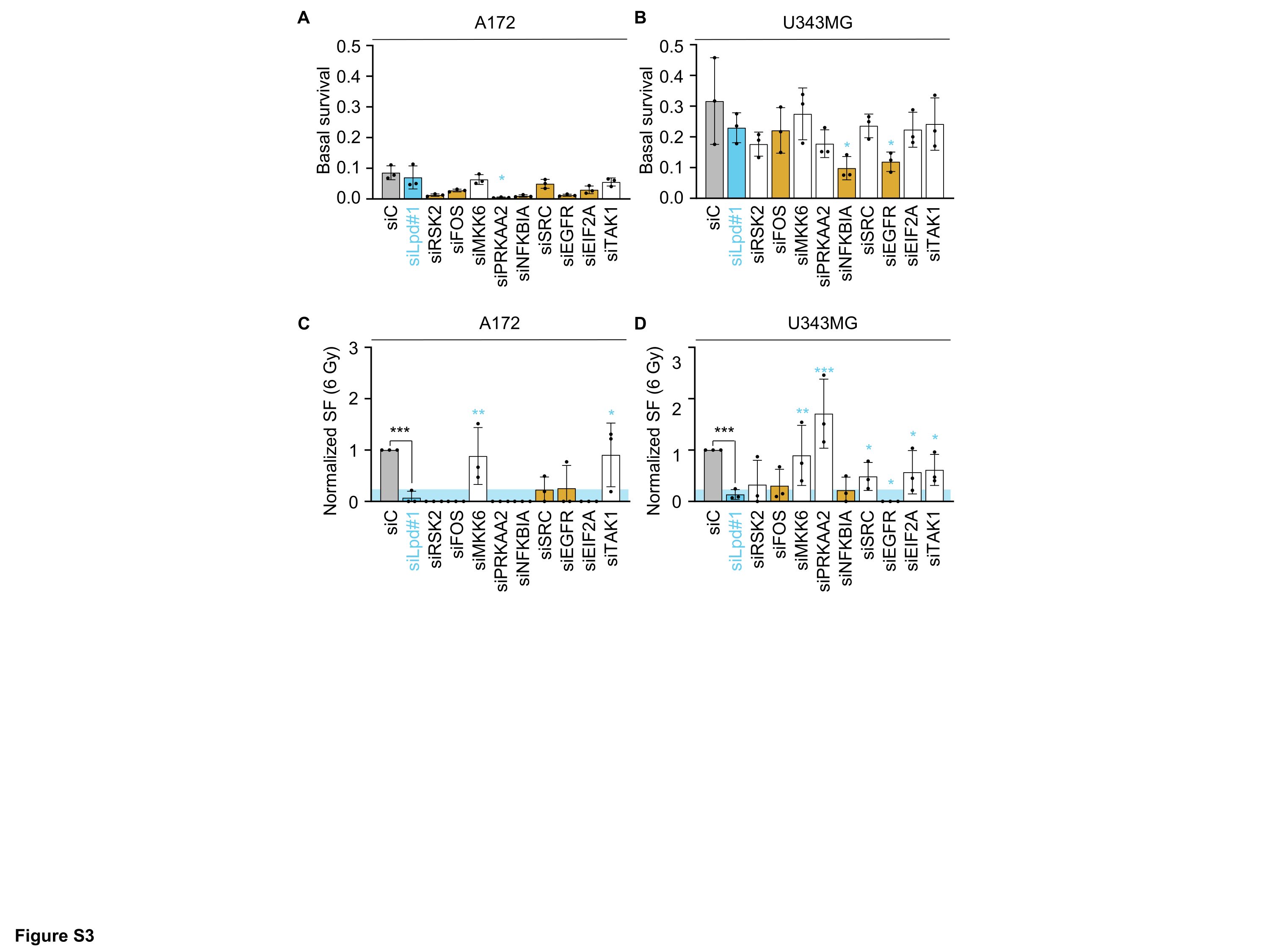

Supplement: Supplementary file 1 [file cancers-13-05337-s001.zip › cancers-1386069 supplementary/Moritz et al_Figure S3.tif]

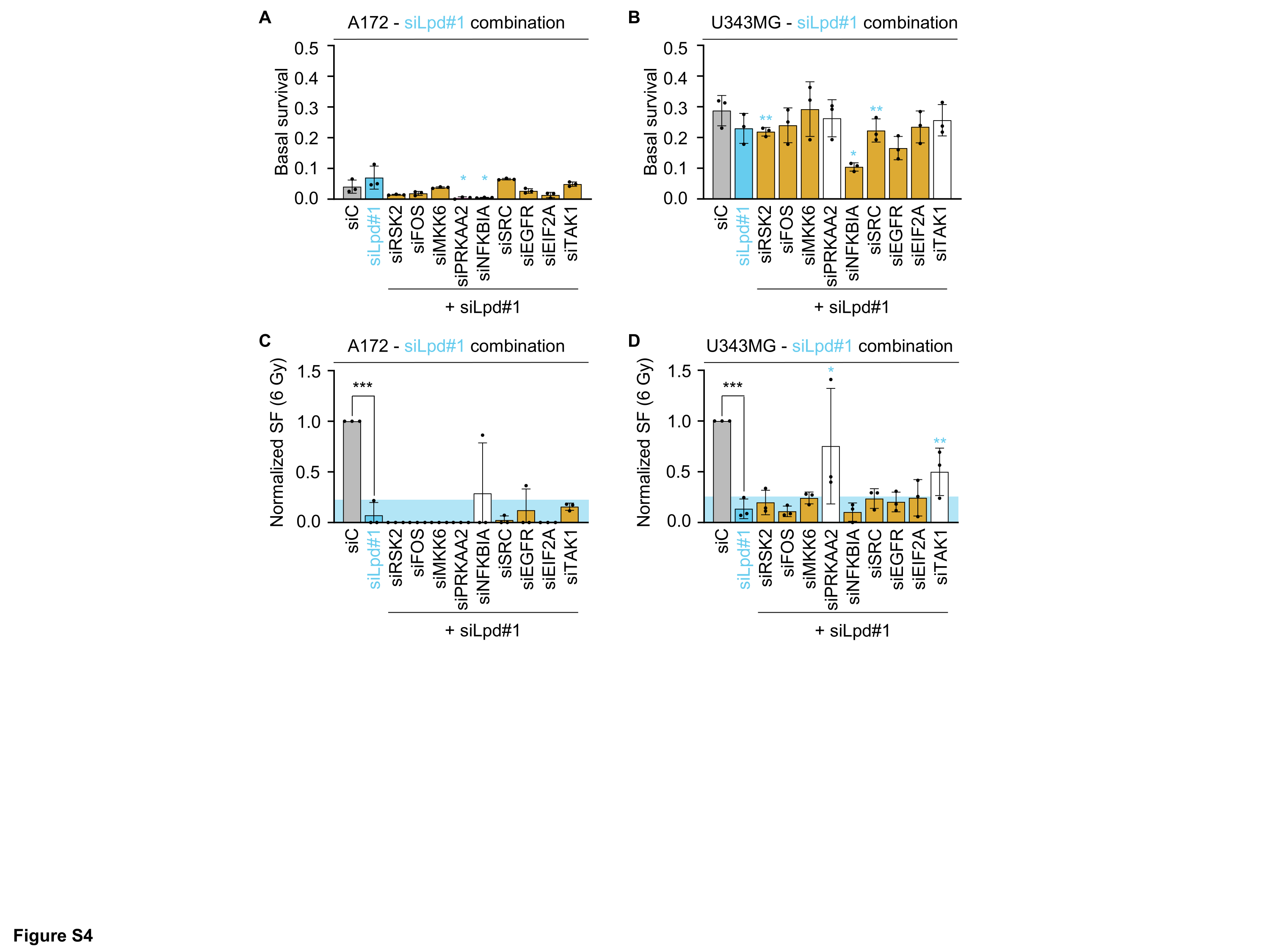

Supplement: Supplementary file 1 [file cancers-13-05337-s001.zip › cancers-1386069 supplementary/Moritz et al_Figure S4.tif]

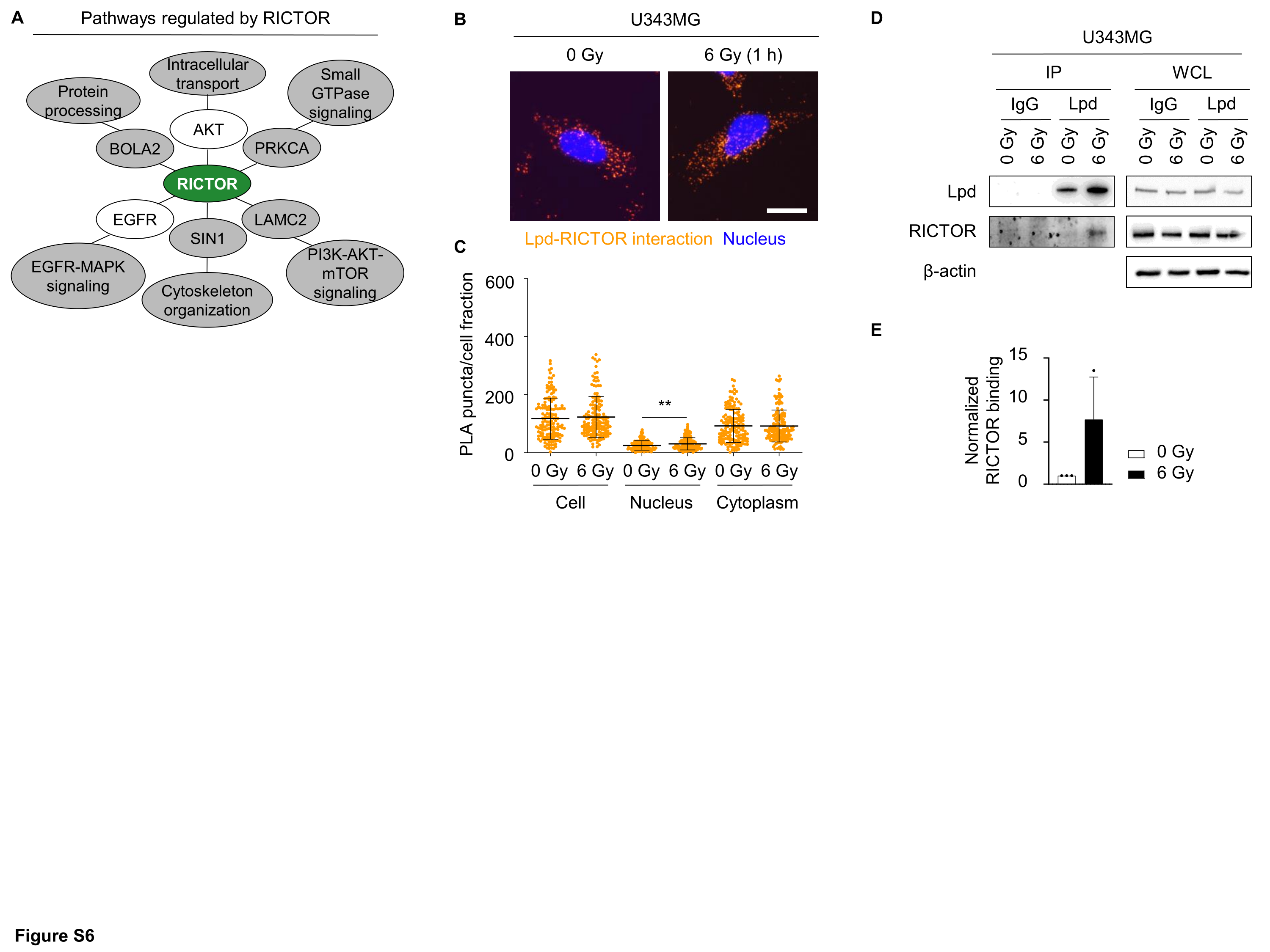

Supplement: Supplementary file 1 [file cancers-13-05337-s001.zip › cancers-1386069 supplementary/Moritz et al_Figure S6.tif]

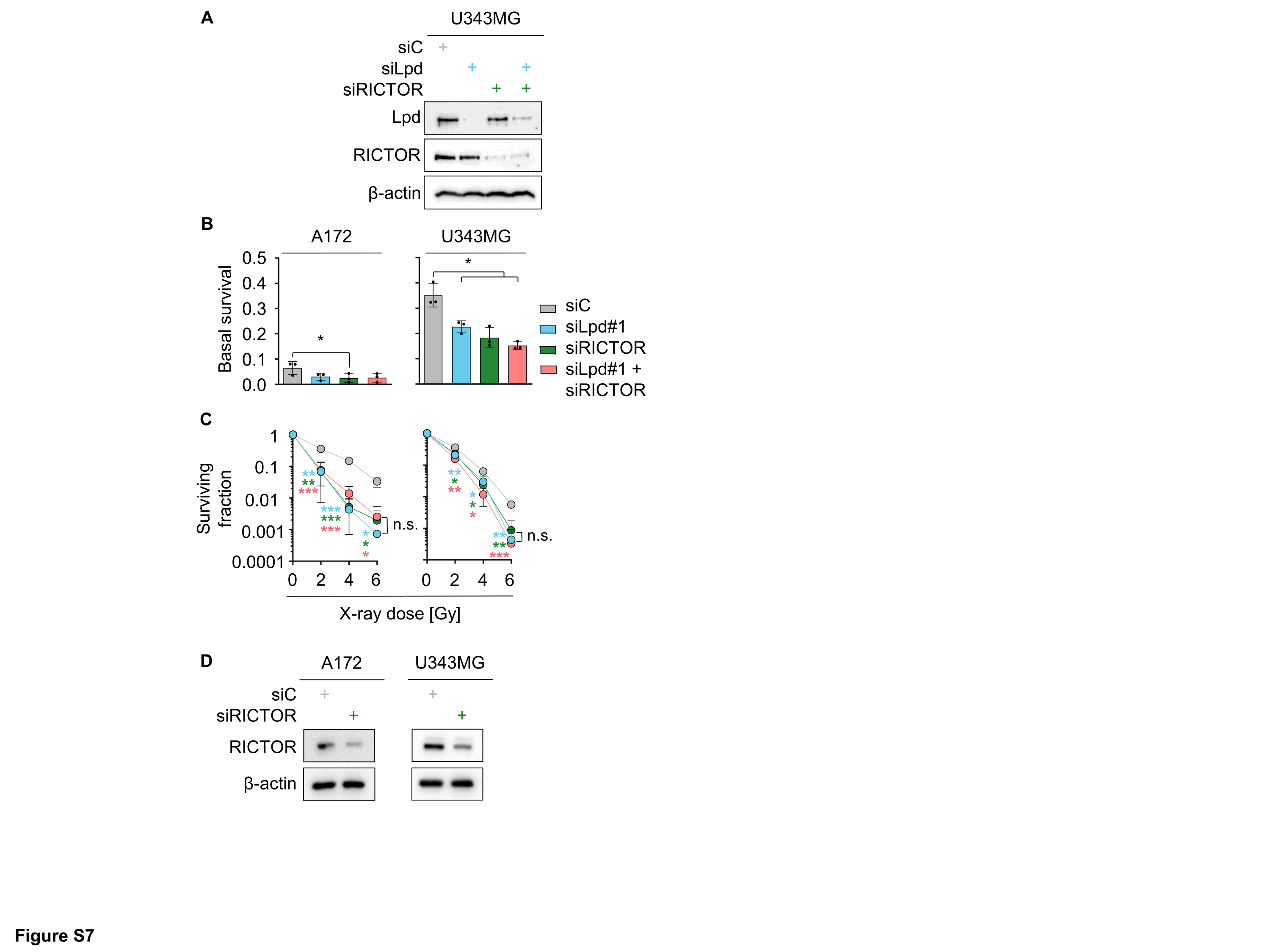

Supplement: Supplementary file 1 [file cancers-13-05337-s001.zip › cancers-1386069 supplementary/Moritz et al_Figure S7.tif]

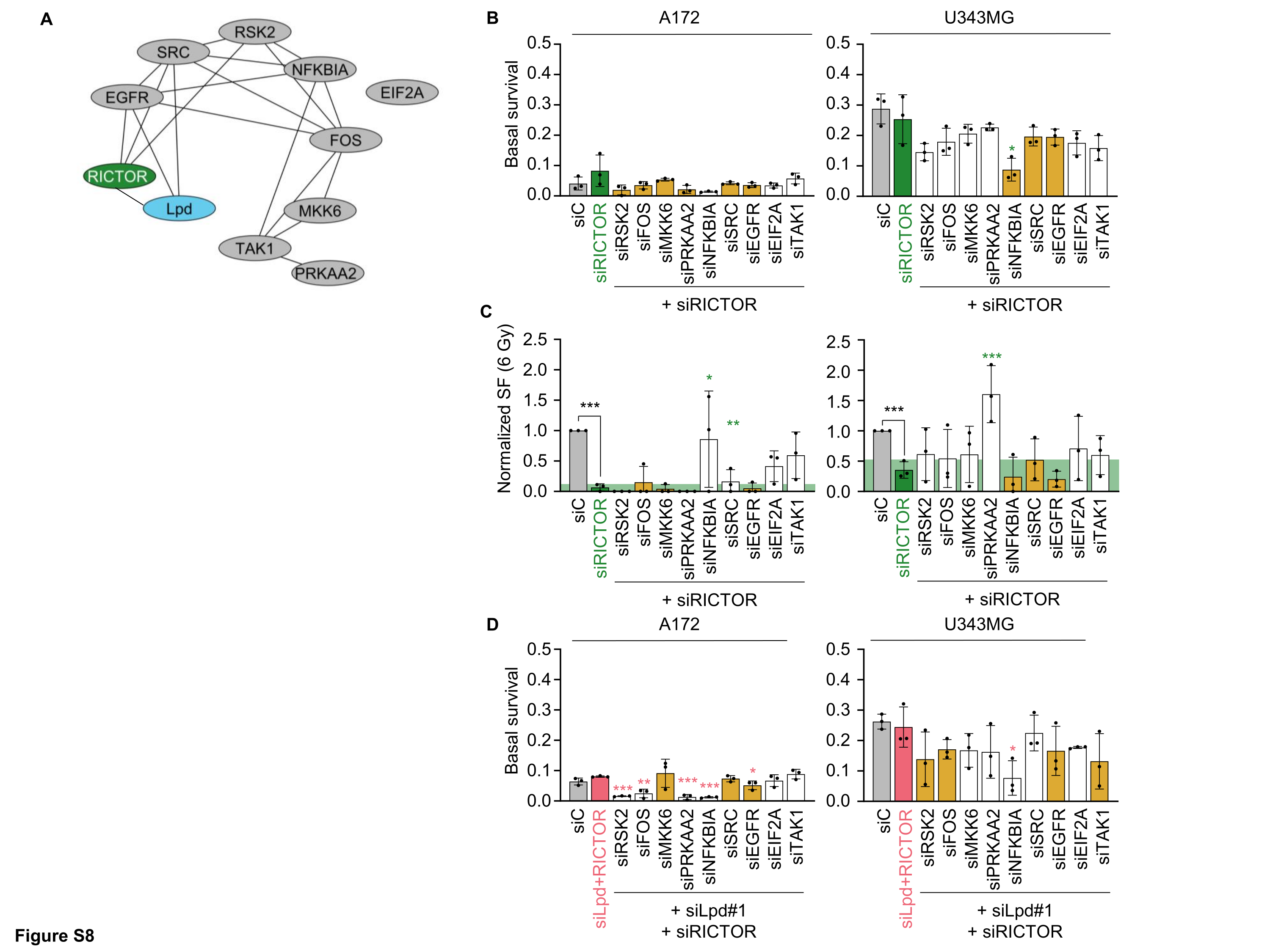

Supplement: Supplementary file 1 [file cancers-13-05337-s001.zip › cancers-1386069 supplementary/Moritz et al_Figure S8.tif]

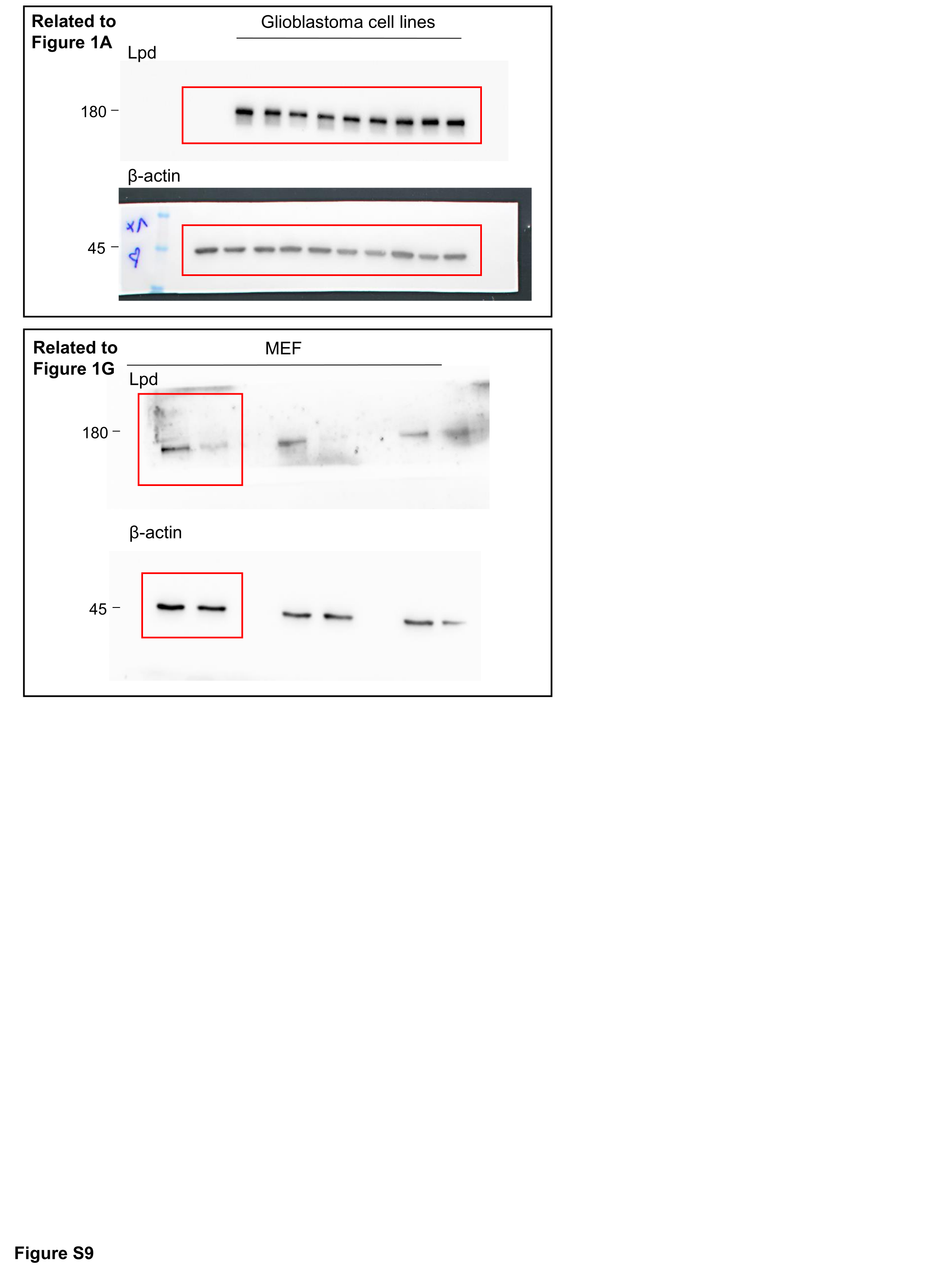

Supplement: Supplementary file 1 [file cancers-13-05337-s001.zip › cancers-1386069 supplementary/Moritz et al_Figure S9.tif]
